# Blockchain enabled collective and combined deep learning framework for COVID19 diagnosis

**DOI:** 10.1038/s41598-025-00252-7

**Published:** 2025-05-13

**Authors:** Sudhakar Periyasamy, Prabu Kaliyaperumal, Manikandan Thirumalaisamy, Balamurugan Balusamy, Thenmozhi Elumalai, Veerpratap Meena, Vinay Kumar Jadoun

**Affiliations:** 1 School of Computer Science and Engineering, Galgotias University, 203201 Delhi NCR, India; 2 Department of CSBS, Rajalakshmi Engineering College, 602105 Chennai, India; 3 Shiv Nadar University, 201314 Delhi NCR, India; 4 Department of Information Technology, Panimalar Engineering College, 600123 Chennai, India; 5 Department of Electrical Engineering, National Institute of Technology Jamshedpur, 831014 Jharkhand, India; 6 Department of Electrical & Electronics Engineering, Manipal Institute of Technology, Manipal Academy of Higher Education, Manipal, Karnataka India

**Keywords:** Blockchain technology, Combined learning paradigm, Diagnostic techniques, Hybrid capsule learning network, Predictive modeling, Ensemble learning, Engineering, Biomedical engineering

## Abstract

The rapid spread of SARS-CoV-2 has highlighted the need for intelligent methodologies in COVID-19 diagnosis. Clinicians face significant challenges due to the virus’s fast transmission rate and the lack of reliable diagnostic tools. Although artificial intelligence (AI) has improved image processing, conventional approaches still rely on centralized data storage and training. This reliance increases complexity and raises privacy concerns, which hinder global data exchange. Therefore, it is essential to develop collaborative models that balance accuracy with privacy protection. This research presents a novel framework that combines blockchain technology with a combined learning paradigm to ensure secure data distribution and reduced complexity. The proposed Combined Learning Collective Deep Learning Blockchain Model (CLCD-Block) aggregates data from multiple institutions and leverages a hybrid capsule learning network for accurate predictions. Extensive testing with lung CT images demonstrates that the model outperforms existing models, achieving an accuracy exceeding 97%. Specifically, on four benchmark datasets, CLCD-Block achieved up to 98.79% Precision, 98.84% Recall, 98.79% Specificity, 98.81% F1-Score, and 98.71% Accuracy, showcasing its superior diagnostic capability. Designed for COVID-19 diagnosis, the CLCD-Block framework is adaptable to other applications, integrating AI, decentralized training, privacy protection, and secure blockchain collaboration. It addresses challenges in diagnosing chronic diseases, facilitates cross-institutional research and monitors infectious outbreaks. Future work will focus on enhancing scalability, optimizing real-time performance and adapting the model for broader healthcare datasets.

## Introduction

The COVID-19 pandemic, a global health crisis with unprecedented medical and socio-economic impacts, has devastated many lives worldwide. At the core of this disaster is the severe acute respiratory syndrome coronavirus, commonly known as SARS-CoV, which affects a sizable portion of the world’s population^1,2^. Notably, India ranks second globally in confirmed COVID-19 cases, with over 44,995,802 reported cases, and is third highest in terms of fatalities caused by the virus. Because diseases are developing exponentially, governments and healthcare systems are ill-equipped to deal with the rising number of ailments^3,4^. Organizations like the World Health Organization (WHO) and the Centers for Disease Control and Prevention (CDC) emphasize that COVID-19 remains a public health concern, particularly for vulnerable populations. This awful situation has made it extremely difficult for physicians to accurately and promptly identify individuals who are COVID-19 positive, mostly because there are not enough testing models available. Important steps in the COVID-19 diagnostic process include pathogenic testing, clinical symptoms, epidemiological history, and computed tomography (CT) scans. However, CT scans of COVID-19 patients often reveal visual symptoms similar to other lung disorders, even with the use of several radiological modalities^3,5^. This complicates diagnosis, particularly considering the wide range of symptoms experienced by patients and the rapid transmission of the disease^6,7^. To facilitate accurate diagnosis, hospitals may need to share data on COVID-19 patients. However, exchanging this sensitive data securely without compromising user privacy is challenging^8,9^. The legitimate concerns about privacy in healthcare organizations that plague the existing research environment make it challenging to manage the complexity of global model training and data transfer. It is essential that AI approaches be enhanced in light of these challenges in order to provide collaborative learning while maintaining user privacy. In order to combat COVID-19 more effectively, it is necessary to find innovative ways to overcome privacy concerns and data-sharing barriers^10–13^.

### Motivation and problem statement

Our investigation is driven by the urgent global need for state-of-the-art diagnostic tools to precisely identify and treat the widespread consequences of the COVID-19 pandemic^14,15^. As highlighted in the introduction, countries like India continue to face significant challenges due to the exponential rise in cases and fatalities, emphasizing the critical importance of innovative diagnostic frameworks. Recent research from the WHO indicates that the virus mostly attacks the lungs, causing long-term harm even after recovery^16,17^. Motivated by this, we are attempting to classify intricate lung patterns caused by COVID-19 in order to enhance the accuracy of diagnosis and prevent errors made by experienced radiologists. However, developing a reliable deep learning model is challenging due to the need for secure sharing of sensitive data, which raises privacy concerns^18^. This issue is exacerbated by the difficulty of fine-tuning predictive models, the scarcity of comprehensive training data, and the complexity of diagnosing intricate lung patterns from CT scans. Collectively, these factors highlight the need for a collaborative framework that preserves privacy while ensuring accurate COVID-19 diagnosis. The proposed approach tackles these key challenges by creating a solution that can be applied to a wider range of healthcare contexts. The main challenge is facilitating collaborative model training while protecting patient confidentiality, especially regarding sensitive CT scan data. Existing diagnostic methods have notable limitations: centralized AI models demand significant computational resources, pose privacy concerns due to centralized data storage, and often suffer from misclassification due to the visual similarities between COVID-19 and other pulmonary diseases. Additionally, these models face scalability issues, making them less suitable for cross-institutional use. Our CLCD-Block framework combines blockchain technology with a collaborative learning approach and ensemble learning methods for the development of a global model. Decentralized model training maintains privacy by securely exchanging model weights instead of raw data through a blockchain-based system. Capsule Networks manage complex feature extraction^19^, Extreme Learning Machines (ELM) provide strong classification capabilities, and ensemble learning combines decentralized models to create a precise and adaptable global model. This approach facilitates secure and scalable collaboration among healthcare institutions, tackling critical challenges related to privacy, accuracy, and computational efficiency.

Although the proposed CLCD-Block framework is specifically designed to tackle the challenges of COVID-19, its structure and methodology are applicable to a wider range of use cases. Utilizing blockchain for secure collaboration and advanced AI methods for accurate diagnosis, the system provides a flexible solution to a wide range of medical challenges. This encompasses the diagnosis of chronic diseases, the management of global health emergencies, and the facilitation of secure data sharing between institutions while maintaining data privacy. This adaptability guarantees that the framework remains applicable to both emerging and re-emerging diseases, along with other crucial healthcare scenarios.

### Objectives

In essence, our goal is to create a combined architecture driven by blockchain that addresses the key challenges identified in current diagnostic models, including privacy, accuracy, and scalability. By integrating capsule networks as a feature extraction technique and Extreme Learning Machines (ELM) as a classifier, our approach enhances the capacity to recognize COVID-19 indicators in various CT scans while permitting secure data transfer across institutions. This innovative combination not only significantly improves diagnostic accuracy but also overcomes the limitations of centralized models, such as high computational demands and privacy concerns, ensuring a reliable and privacy-preserving diagnostic solution in the ongoing fight against COVID-19.

### Key contributions

This research work has three main contributions:

- An algorithm combining ELM and capsule networks is used to detect and classify COVID-19 patterns derived from CT images sourced from various locations. This combination addresses the limitations of conventional deep learning techniques by offering improved feature extraction and higher classification accuracy, thereby minimizing false positives and false negatives.
- An extensive experimental validation showing the superiority of our proposed algorithm over existing deep learning techniques. The results showcase accuracy exceeding 97%, highlighting the model’s effectiveness in real-world COVID-19 diagnostic scenarios and establishing its practical relevance.
- A blockchain-enabled system for secure data collection and sharing, utilizing collaborative learning to ensure privacy during global model training. This approach addresses critical challenges in collaborative learning by preserving data confidentiality while enabling efficient and scalable cross-institutional collaboration.

### Impact statement

Our research presents the Combined Learning Collective Deep Learning Blockchain Model, designed to improve COVID-19 diagnosis by integrating blockchain with a combined learning paradigm. This model ensures secure data distribution and minimizes computational demands while achieving high diagnostic accuracy. The CLCD-Block system gathers data from various healthcare institutions and leverages a hybrid capsule learning network for accurate detections. Empirical validation using diverse lung CT image datasets demonstrates superior performance, achieving accuracies exceeding 97% in detecting COVID-19. Additionally, the blockchain-enabled system facilitates secure data sharing through decentralized model weight updates rather than direct data transmission, thereby preserving user privacy. Comparative analysis against existing deep learning models shows that our approach not only achieves higher accuracy but also addresses challenges related to data security, privacy, and computational efficiency. This innovative integration of blockchain technology with deep learning offers a robust and privacy-preserving diagnostic solution, which is critical for enhancing the effectiveness of pandemic response efforts.

### Organization of the study

This research is structured into five sections to systematically present the proposed approach and its validation:

- Section Introduction: Covers the motivation, objectives, key contributions, impact statement, and organization of the study, setting the context for the research and highlighting its significance.
- Section Related works: Provides a comprehensive review of existing studies and approaches related to COVID-19 diagnosis, blockchain integration, and ensemble learning, identifying research gaps addressed in this work.
- Section CLCD-Block model: Elaborates on the proposed methodology, including data preprocessing, model architecture, and training processes. It introduces the materials and methodologies used, details the data normalization techniques, and describes the ensemble capsule-based model training. This section also covers the integration of capsule networks and extreme learning machines, global model training, and the blockchain-based data sharing process.
- Section Experimental results: Presents the experimental setup, including dataset specifications, evaluation metrics, and complexity analysis. This section also discusses the results obtained, comparative performance analysis with existing methods, and limitations of the proposed approach.
- Section Conclusion and future directions: Summarizes the key findings, emphasizes the contributions of the study, and outlines future research possibilities and improvements.

## Related works

When Scientists have looked into a variety of cutting-edge techniques in the field of medical image analysis in an effort to enhance early sickness detection^14,20,21^. Data transfer, virtual monitoring, stage classifications, e-treatment, and prediction were among the subjects of their study^22^. It’s interesting to note that the study employed SVM, DT, KNN, and ANN among other supervised learning classifiers in the context of medical imaging^23,24^. Building on this foundation, M.A. Talukder et al.^25^ aims to improve COVID-19 detection using advanced deep learning methods, with a particular focus on the EfficientNet architecture. The research underscores the difficulties of data scarcity during the pandemic and stresses the significance of transfer learning, enabling models to utilize existing knowledge from large datasets to enhance COVID-19 detection performance. The authors conducted an extensive assessment, demonstrating that their optimized model achieved high accuracy in detecting COVID-19 from X-ray images. The results indicate that this approach has the potential to enhance early detection techniques, ultimately aiding in improved healthcare diagnostics and more effective public health management during the current pandemic. The authors suggest that future research could improve the models by integrating feature extraction and selection techniques, along with broadening the analysis to encompass additional imaging modalities, like MRI and CT scans. Similarly, the study^26^ concentrates on enhancing diabetes detection through machine learning (ML) methods, highlighting the creation of an optimized data preprocessing pipeline to improve the quality of diabetes-related datasets. Major contributions involve tackling imbalanced datasets via random oversampling, mitigating overfitting using k-fold cross-validation, and performing thorough experimental validation on a variety of datasets. The study demonstrated a notable average accuracy of 95.5% in predicting diabetes, outperforming current methods. The authors emphasize the importance of additional research in feature selection, ensemble methods, and deep learning techniques to improve the reliability and effectiveness of their approach in healthcare applications. The significance of blockchain technology was underlined, particularly in light of its ability to facilitate global data transfer and public access to medical records^27^. The authors concluded that these technological developments are critical to the state of medical image transmission today and have a significant impact on the healthcare industry. However, the study also point out that patient privacy issues were a challenge for the COVID-19^28,29^. To overcome these privacy restrictions, the article provided a comprehensive solution in the form of privacy architecture. This innovative approach uses combined learning and blockchain technology to safeguard individual privacy, enhance public communication, and open up new channels for the exchange of crucial COVID-19 data. The resistance of the suggested architecture to potential privacy and information security breaches was the primary factor leading to its resilience^30^. In an attempt to make a similar contribution to the field of medical imaging, another study presented PriMIA, an open-source software platform based on combined learning. PriMIA was developed specifically to handle several sources of pediatric radiological data for classification requirements^31^. To classify different stages of heart illness, this design featured a deep convolutional neural network (DCNN), which was trained on a database of pediatric chest X-ray pictures. Moreover, the application of blockchain technology produced a novel paradigm with a focus on security. This technology aimed to identify rogue nodes as a strategy to improve the reliability and accuracy of decision-making within a network by ensuring that nodes can effectively validate their inputs and collaborate to generate trustworthy outcomes^32^. The proposed approach attempted to reduce communication costs during combined learning while simultaneously successfully thwarting two prevalent risks known as “free-riding attacks” and “model poisoning attacks.” Together, these developments have a major impact on the ever-changing field of medical imaging and healthcare data security^33^. In addition to addressing present issues, the incorporation of cutting-edge technologies like blockchain and combined learning opens the door for safer and more private methods of managing medical data and diagnosing diseases^31^. In the realm of decentralized combined learning, a concept called “BlockFL” was introduced with the intention of expanding the usage of combined learning while decreasing the single point of failure that is characteristic of traditional combined learning by including local training outcomes into the verification process. FLchain provides a health-focused, publicly audited, audited combined learning environment that is centralized^34,35^. This approach replaces the conventional central coordinator of combined learning with blockchain technology, providing a fresh perspective on trust and motivation in combined learning. Examining the concept of channels, blockchain architecture with global models was presented^36^. This channel-specific ledger-based approach tracked and records model parameters using blockchain-like technology, ensuring collaborative and decentralized data preservation while enhancing transparency and trust in data management. Combined learning and crypto currencies were employed, together with the establishment of a database that functions outside the primary blockchain, this system efficiently tracks gradients and manages rewards while effectively addressing privacy and security concerns^30^. Moreover, a personalized combined learning algorithm was recommended for patients with COVID-19. The pre-trained neural network with the aim of forecasting the severity of death and providing e-treatment using images from erroneous chest X-rays of patients. However, there may be issues with the derived predictor when working with huge datasets. Taken as a whole, these developments support ongoing research into novel architectures and algorithms at the intersection of blockchain and combined learning, addressing important issues in privacy, data security, and medical applications^37^. In parallel endeavors, researchers have presented the COVID-CAPS approach to address the challenges presented by small datasets. The inadequacies of CNN-based models in the detection of COVID-19-positive patients from X-ray images are the main emphasis of this model, which was developed by^38,39^. It’s interesting to note that, when its parameters are set for optimal results, the COVID-CAPS model outperforms traditional networks. In a distinct area of study, He and colleagues conducted an experimental study examining automated Combined learning (AutoFL) using a Combined NAS (FedNAS) algorithm and the neural architecture search (NAS) technique^40^. By facilitating the seamless incorporation of updates from local machine learning models, AutoFL aims to enhance the quality and performance of these models. The study’s noteworthy findings imply that local machine learning models’ default parameters might not be suitable for a combined environment, especially for clients that lack a unique identity (non-IID)^41–44^. These related works have been extremely beneficial to the field of machine learning techniques. The COVID-CAPS model highlights efforts to refine and improve machine learning approaches, particularly with reference to healthcare and sickness detection, by demonstrating possibilities for improving the capacity to recognize COVID-19 occurrences from X-ray. Simultaneously, the study of automated combined learning produces important insights for enhancing the effectiveness of local machine learning models, which may result in innovations in decentralized and collaborative learning techniques^45^.

Recent studies in medical image analysis have explored various supervised and deep learning techniques to enhance early disease diagnosis, especially during the COVID-19 pandemic. Models like EfficientNet and COVID-CAPS improved performance on limited datasets, while transfer learning addressed data scarcity. Efforts in privacy-preserving solutions using federated learning (FL) and blockchain, such as PriMIA and FLchain, enabled secure, decentralized medical data sharing. However, most research has focused on X-ray and MRI data, with limited emphasis on CT scan analysis despite its superior diagnostic detail. Existing approaches also face challenges like data heterogeneity, lack of model generalization, and privacy concerns in collaborative learning environments. These gaps highlight the need for more secure, adaptive, and robust frameworks specifically designed for CT scan-based diagnosis, integrating advanced FL architectures and privacy-preserving techniques to ensure accurate and trustworthy detection across diverse healthcare settings. The following table 1 provides a comprehensive summary of recent advancements in medical image analysis and disease detection techniques, highlighting key contributions, methodologies, and observed outcomes

**Table 1:** Summary of related works on medical image analysis and disease detection techniques.

Methodology	Problem addressed	Key findings	Planned improvement
EfficientNet, Deep Learning^25^	COVID-19 Detection from X-ray Images	Achieved high accuracy through transfer learning to address data scarcity during the pandemic.	Integrating feature extraction and selection techniques, exploring MRI and CT scan data.
Machine Learning (SVM, DT, KNN, ANN), Data Preprocessing^26^	Diabetes Prediction	Optimized data preprocessing to tackle data imbalance, achieved 95.5% accuracy.	Investigating feature selection, ensemble methods, and deep learning techniques.
Blockchain Technology^27^	Data Privacy and Secure Data Transfer	Enhanced public access to medical records with privacy architecture to address security concerns.	Improve privacy mechanisms and integrate with advanced AI models.
Deep CNN, Blockchain^31^	Pediatric Radiology	Developed open-source software for pediatric chest X-ray classification, addressing privacy and security through blockchain.	Enhance communication efficiency and address free-riding and model poisoning attacks.
Blockchain with Combined Learning^35^	Federated Learning	Reduced single-point failures by using decentralized blockchain for model verification.	Implement personalized models for different healthcare settings.
Combined NAS (FedNAS), Neural Architecture Search^40^	Federated Learning Automation	Improved model integration from local ML models, highlighting the challenge of non-IID data.	Improve compatibility and optimization for non-IID client data.
Capsule Network (CapsNet)^38^	COVID-19 Detection from X-ray Images	Outperformed CNN-based models on small datasets, offering enhanced robustness and performance.	Fine-tune model hyperparameters and explore multi-modal data fusion.
Automated Combined Learning, FedNAS^41^	Federated Learning	Demonstrated the limitations of default local ML model parameters in combined learning environments.	Develop adaptive parameter optimization strategies.
Blockchain and AI Integration^14^	Vaccine Distribution and Monitoring	Enhanced traceability and monitoring of vaccine distribution during the pandemic.	Integrate predictive analytics for proactive decision-making.
Machine Learning (SVM, DT, KNN, ANN)^20^	Parkinson’s Disease Recognition	Compared various ML models for accurate disease recognition, achieving high accuracy with specific algorithms.	Incorporate deep learning models for feature extraction and classification.
Privacy-Preserving Architecture, Blockchain^30^	Privacy and Data Security	Developed a robust architecture to protect data privacy while enabling secure communication.	Integrate lightweight cryptographic methods for faster data processing.
Blockchain with Federated Learning^33^	Secure Medical Imaging Transmission	Improved data transmission security and resilience against model poisoning and free-riding attacks.	Optimize communication cost and latency during federated learning.

## CLCD-Block model

**Fig. 1:**
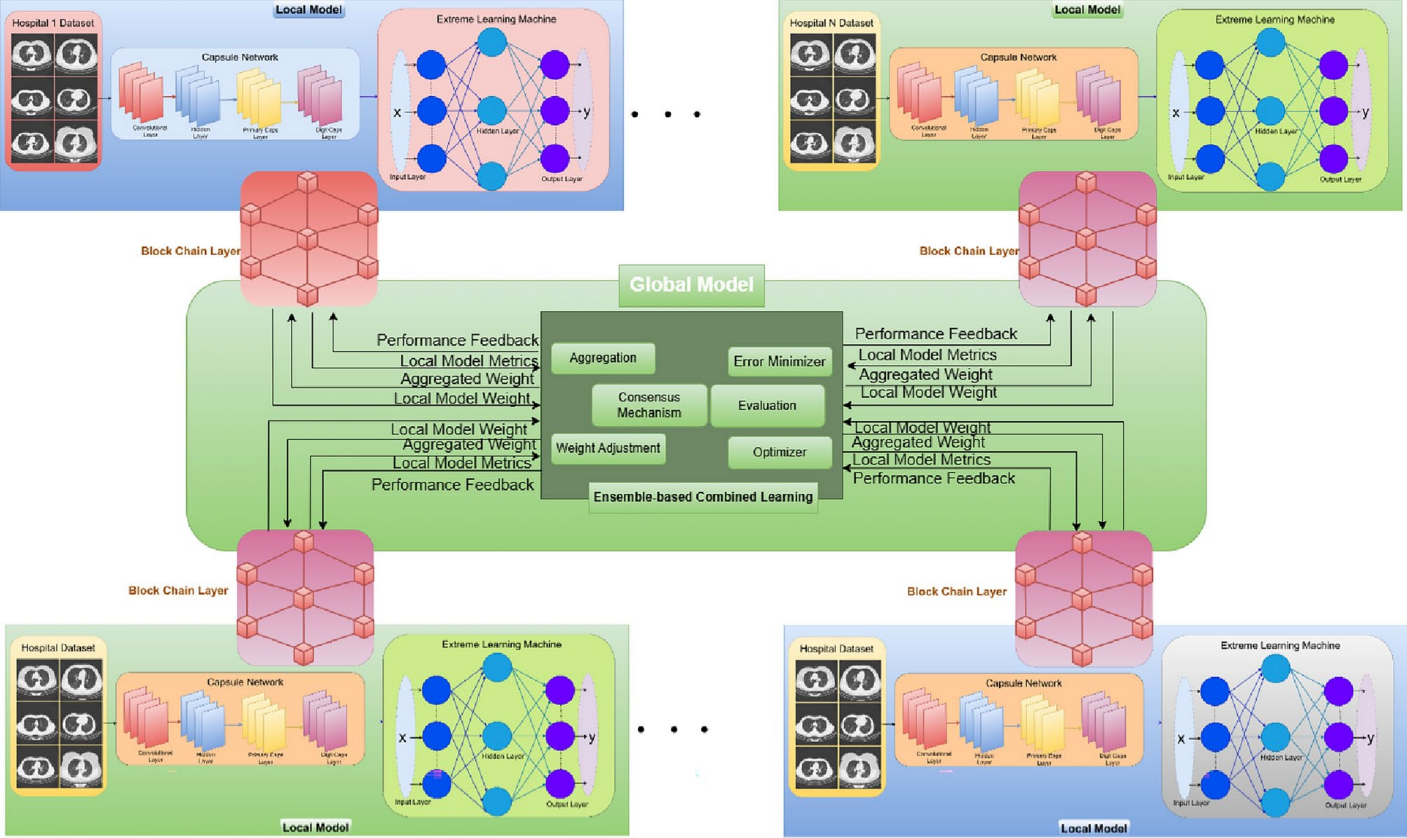
Framework of the proposed CLCD-block for effective prediction and diagnosis of COVID-19 using blockchain-enabled combined learning.

**Fig. 2:**
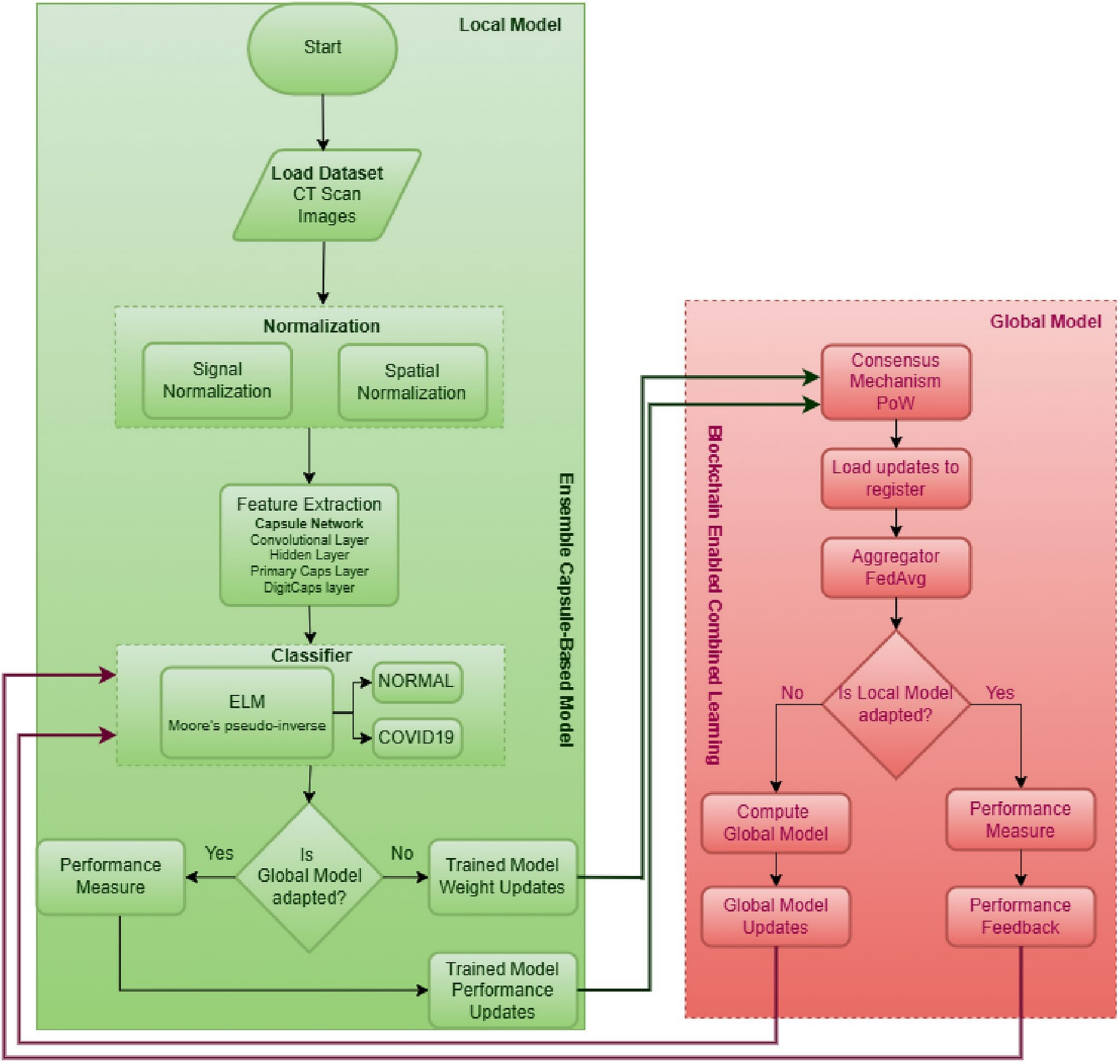
Flowchart of the proposed CLCD-block for enhanced prediction and diagnosis of COVID-19 using blockchain-enabled combined learning approach.

The challenge of sharing patient data between healthcare organizations due to privacy concerns impedes the successful collaboration and use of deep learning models for sickness identification. A large amount of data is needed for deep learning models, which raises processing times, complicates problems, and often results in performance degradation. In order to address real-time challenges, this study proposes the CLCD-Block model, which is meant for global model sharing and training. The recommended structure is depicted in Fig. 1, and the step-by-step procedure of the proposed CLCD-Block framework is demonstrated in Fig. 2.

The coordinated collection of data from several hospitals using various types of CT scanners is made possible by the CLCD-Block concept. The initial stages are to normalize the data using spatial and signal normalization. Using deep learning techniques-specifically, an ensemble capsule network-to recognize COVID-19 patterns in lung CT scan data is the next stage. The image segmentation and ensemble capsule network training methods enhance generalization over earlier learning models. Next, COVID-19 classification is done using extreme learning machines (ELMs), which are neural networks with a self-tuning hidden layer. Global model creation uses combined learning approaches to solve privacy issues. Using a public network, this method collects data, trains a collective adaptive model, and disseminates the model. Most significantly, combined learning uses blockchain technology to exchange just weights and gradients, protecting patient privacy in hospitals. The secure transfer of data between several entities is ensured by this decentralized design, all while protecting patient privacy^32,38^. To give a more detailed explanation, the proposed framework comprises data collection from various medical healthcare facilities, model training for COVID-19 image segmentation and classification using a hybrid capsule learning network, and sharing the hybrid model with other parties via Combined learning and blockchain, all while preserving and respecting organizational privacy. This research paper offers a comprehensive strategy that addresses the technological challenges related to the use of deep learning models and emphasizes the necessity of privacy preservation in healthcare data collaboration.

### Materials and methodologies

Clinical diagnosis now requires the incorporation of artificial intelligence. Significant amounts of data are required for the training of deep learning algorithms, which emphasizes how crucial it is to gather reliable data in order to evaluate the suggested model. The model in this work was trained using a range of heterogeneous CT imaging data sources; datasets 1, 2, 3 and 4 were the datasets used for model assessment.

#### Dataset 1

**Fig. 3:**
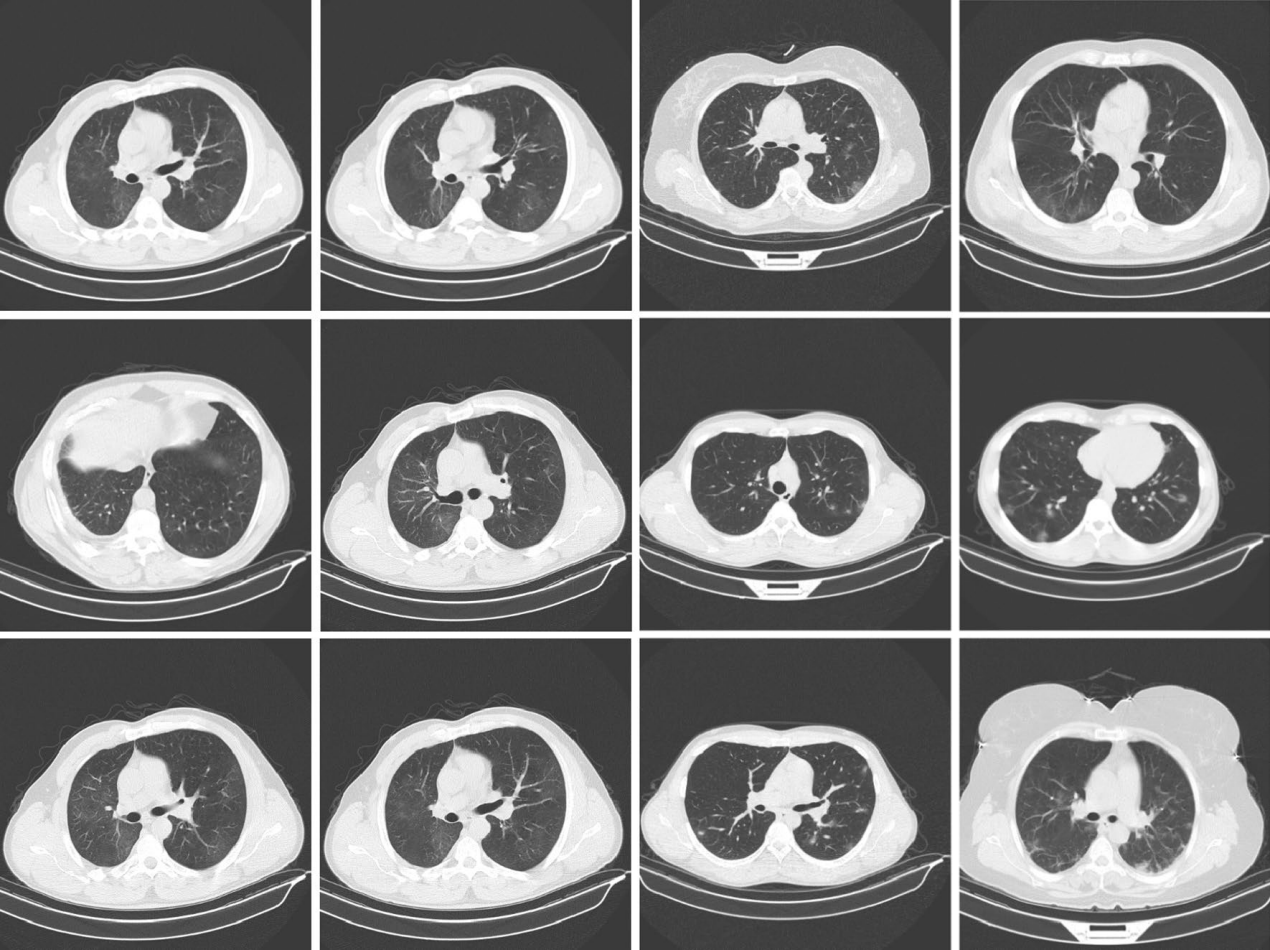
Sample lung CT scan images from dataset 1.

Three universities’ 34,006 CT scan slices, or 89 patients, make up the first dataset^46^. 28,395 of these slices are connected to people who test positive for COVID-19.The data was collected from six different scanners and includes CT scan slices for each of the 89 individuals. In this cohort, 68 individuals were found to be positive for the COVID-19 virus, whereas 21 individuals were identified as negative. Table 2 below illustrates the distribution of the CT image dataset, while samples of lung CT scans are presented in Fig. 3.

#### Dataset 2

People who underwent unenhanced chest CT scans and had COVID-19 infections verified are the subject of this second dataset^47^. Patients frequently indicated coexisting illnesses such as diabetes, coronary heart disease, emphysema or interstitial pneumonia, hypertension, or emphysema. Images of individuals who tested positive for COVID-19 by “Reverse Transcription Polymerase Chain Reaction” (RT-PCR) and who also had accompanying clinical symptoms were captured in an in-patient setting during the period from March 2020 to January 2021. The data collected as images were acquired using a NeuViz 16-slice CT scanner in “Helical” mode without the use of intravenous contrast. Every image is in the DICOM format, with 512 $\times$ 512 pixels and 16-bit gray-scale.

#### Dataset 3

There are 463 non-COVID-19 CT scans and 349 COVID-19 CT scans from 216 patients in this dataset^48^. These files comprise baseline CT scan images taken at the point of care from patients whose SARS-CoV-2 presence was confirmed by RT-PCR during an outbreak. A normalization technique has been created to modify the CT scan visuals for a combined learning mechanism in light of the various data sources. A summary of the datasets utilized to assess the suggested combined learning model is given in Table 2.

#### Dataset 4

**Fig. 4:**
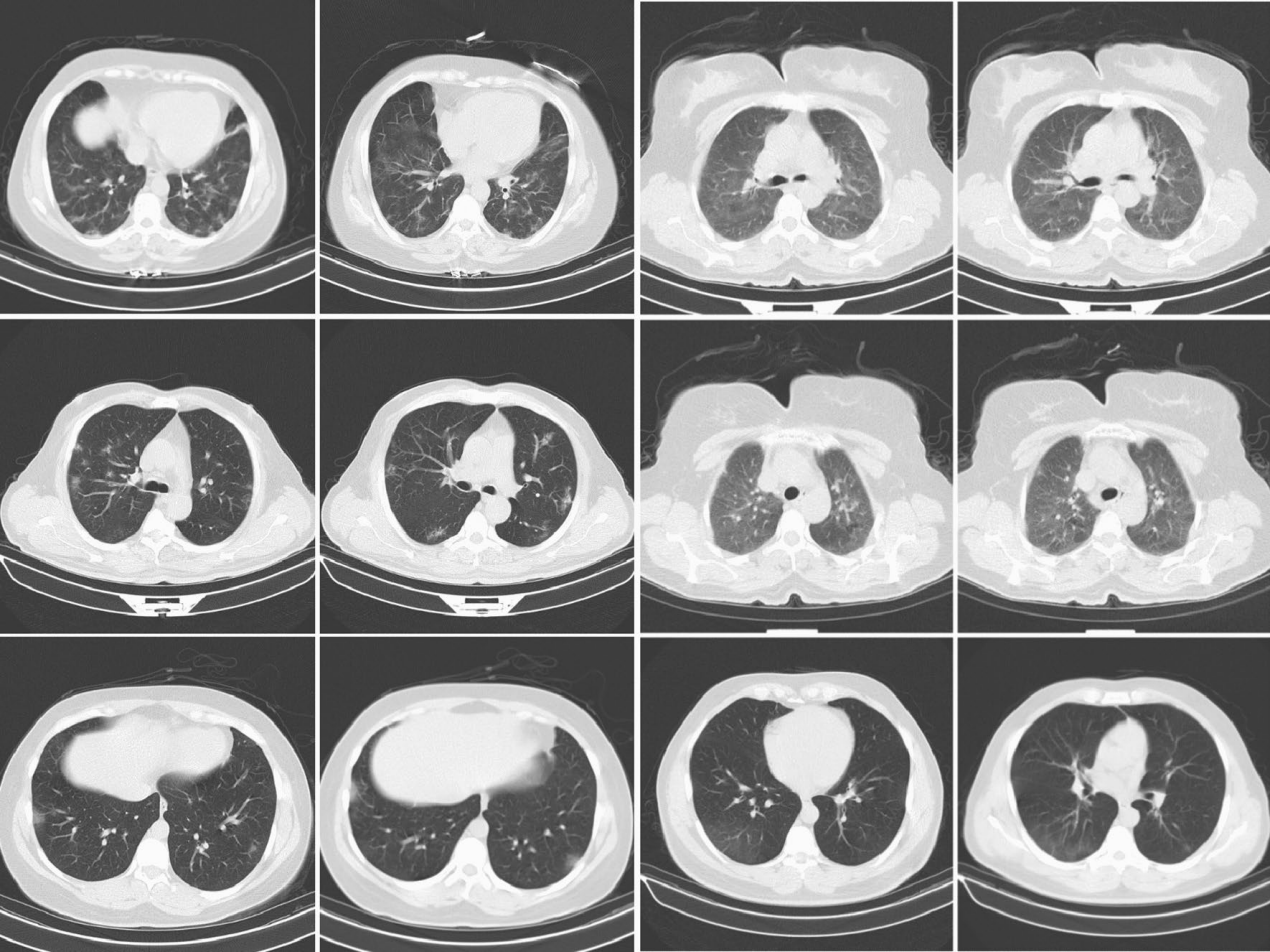
Sample lung CT scan images from dataset 4.

This dataset consists of 425,024 chest CT images categorized into three classes: 71,488 images from 649 COVID-19 positive patients, 42,943 images from 932 pneumonia patients, and 310,593 images from 3,731 normal cases. The chest CT images, sourced from COVIDx CT-3A^49,50^ are used in this study for multiclass classification. The dataset is heterogeneous, with images collected from various countries, age ranges, and genders, all in PNG format. A visual depiction of the sample datasets used in the experiments is shown in Fig. 4. This dataset was selected for its multiclass structure, enabling multiclass classification, in contrast to Datasets 1, 2, and 3, which focus on binary classification for COVID-19 detection.

#### Dataset selection and preprocessing

The selection of datasets was carried out with careful consideration of their relevance, heterogeneity, and availability. The preprocessing steps were implemented to ensure uniformity across diverse data sources, thereby enhancing the robustness of the combined learning models. The selection and preprocessing of datasets were carried out with careful consideration of their relevance, heterogeneity, and availability to ensure the robustness of the combined learning models. The datasets were chosen for their diverse nature and relevance to COVID-19 diagnosis, encompassing both binary and multiclass classification. Emphasis was placed on selecting publicly available datasets containing CT images from various sources, representing a wide range of demographics, medical conditions, and geographical locations. Furthermore, the selected datasets were evaluated for compatibility with deep learning frameworks, maintaining standardized formats such as DICOM and PNG. To ensure uniformity and enhance model performance, several preprocessing steps were undertaken. First, resolution standardization was applied, converting all images to a consistent size of 512 $\times$ 512 pixels. Intensity normalization was also performed, utilizing both signal and spatial normalization techniques to maintain consistency in voxel intensities and image sizes. additionally noise reduction was achieved through Gaussian filtering to minimize noise and improve the quality of the CT images. Format conversion was performed when necessary, transforming DICOM images into PNG format to enhance compatibility. Unlike many deep learning approaches that rely on data-level interventions-such as augmentation, resampling, or synthetic sample generation-to address class imbalance, the proposed Capsule-based Ensemble Learning Framework (CLCD-Block) is designed to handle imbalance intrinsically. Capsule Networks, with their dynamic routing-by-agreement mechanism, effectively preserve critical spatial hierarchies and amplify minority class features during training. This routing mechanism dynamically allocates attention to underrepresented samples, reducing the risk of their being overshadowed in imbalanced scenarios. Furthermore, the inclusion of Extreme Learning Machines (ELM) in the final classification stage enables high-speed generalization without extensive parameter tuning. ELM’s kernel-based transformations and pseudo-inverse computation provide efficient learning from limited minority instances while avoiding overfitting to dominant class distributions. This architecture-driven strategy inherently mitigates class imbalance without relying on traditional balancing techniques such as SMOTE, class weighting, or heavy augmentation, thereby streamlining the learning process while maintaining high diagnostic accuracy.

**Table 2:** Overview of the dataset information utilized in the suggested study.

Datasets	Covid - 19 positives count	Covid - 19 negative count	Image type	Total patients	CT scan image count
Dataset - 1	68	21	Computed axial tomography	89	34,006
Dataset - 2	783	217	Digital imaging and communications in medicine	1000	45,002
Dataset - 3	216	463	Computed axial tomography / digital imaging and communications in medicine	679	3490
Dataset - 4	1581	3731	Computed axial tomography	5312	425,024

### Data normalization

The data normalization approach described in the pertinent literature is used in the proposed study. A strong normalization strategy is essential to maximize the performance of the suggested combined learning models since heterogeneous data is used. Two sorts of approaches are used in the normalizing approach: spatial normalization and signal normalization. The latter is especially designed to handle CT scans. The following clarifies the functions of various normalizing approaches: spatial and signal normalization techniques.

#### Signal normalization technique

The lung window is used by signal normalization to calculate the voxel’s intensity. Radio density readings are expressed in X-units (XU) for each CT scan. Window center (XC) and window width (XW) are two window characteristics that are often used in medical procedures. Equation (1) is used to get the normalized value while taking these window characteristics into account.

1 $$\begin{aligned} S_{\text {norm}} = \frac{(S - XC)}{XW} \end{aligned}$$

Where S is the original picture that was entered, and Snorm is the image that has to have its intensity normalized. A lower bound window size of $(-0.05, 0.5)$ has been selected for this experiment.

#### Spatial normalization technique

The size and resolution of the CT scan visuals are used to determine the use of spatial normalization^51^. As per established norms, all CT scan visuals are standardized to a dimensions of 332 $\times$ 332 $\times$ 512 mm^3^. Every gathered dataset is subjected to the same normalization process, guaranteeing that every image group file adheres to a common format that supports combined learning. Learning and performance as a whole are enhanced by this uniformity.

### Ensemble capsule-based model training

Deep learning frameworks have been increasingly popular recently, mainly because of their effective classification processes and strong feature extraction layers^52–54^. Convolutional neural networks (CNNs) have been the go-to option for image categorization analysis in recent years. Nevertheless, the spatial correlations between features in an image are not sufficiently taken into account by CNN’s pooling layers, which increases computing complexity and may have an effect on classifier performance. The proposed system uses a novel approach to get over the aforementioned problems by combining the benefits of extreme learning machines (ELM) and capsule networks to increase classification accuracy and diagnostic capabilities. This method extracts robust feature maps using capsule networks and employs ELM in place of conventional dense classification layers to yield improved predictions for COVID-19.

### Capsule network

**Fig. 5:**
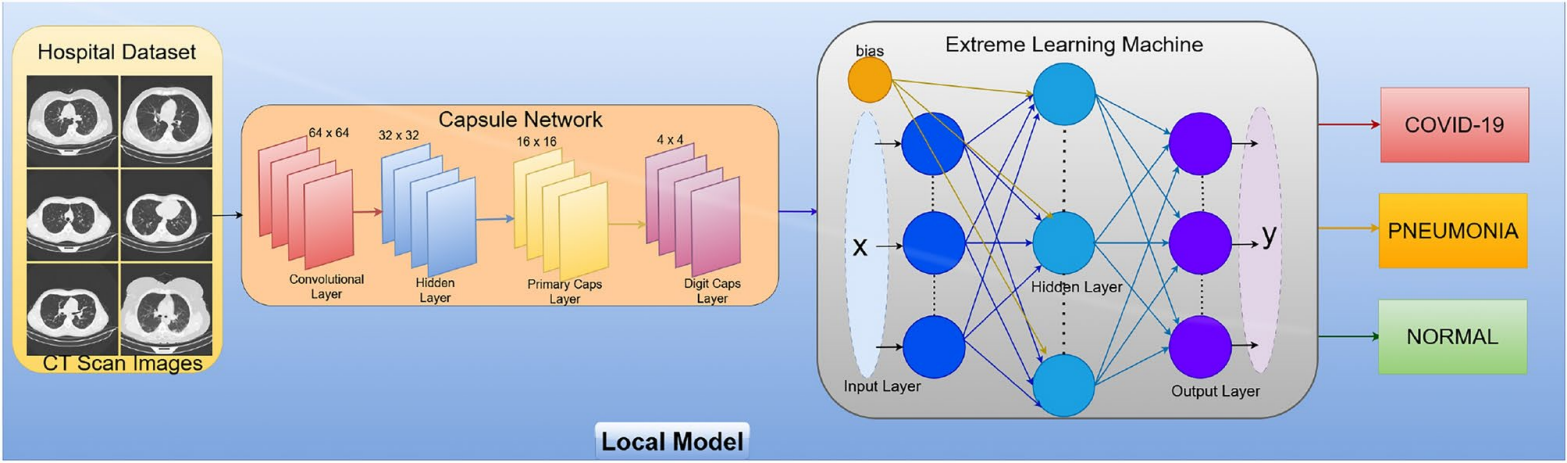
Capsule networks combined with ensemble ELM layers are utilized to enhance the accuracy of feature extraction and classification.

The capsule network consists of four main layers: the convolutional layer, the hidden layer, the Primary Caps layer, and the DigitCaps layer. Together, these layers are able to effectively manage some constraints. The whole design of the recommended training model is displayed in Fig. 5. The capsule networks’ input is the normalized input image, and the process is split into two phases:

1. Likelihood of an entity’s existence.

2. Parameters used during entity instantiation. To illustrate the critical spatial correlations of the image between high and low-level features, Eq. (2) is computed with input vectors denoted as “V,” a weight matrix named “M,” and a component vector called “X.”

2 $$\begin{aligned} Y(i,j)=M (i,j) . X(i,j)*V(j) \end{aligned}$$

Equation (3) is used to find the current capsule “C” by adding together the weighted input vectors.

3 $$\begin{aligned} V(j)= \sum _j Y(j,i)*C (j) \end{aligned}$$

Lastly, Eq. (4) applies non-linearity via the squash function.

4 $$\begin{aligned} Y(i,j)=M_(i,j) X (i,j)* V_j \end{aligned}$$

Until an ideal distribution is reached, the distribution of the “low level capsule to the high-level capsule” is gradually changed based on the findings.

#### Parameters utilized in capsule networks

The network receives a normalized image as input, which is initially passed through a convolutional layer to capture low-level features. Next, the features are forwarded to the hidden layer and the Primary Caps layer, where they are organized into capsules. The capsules represent both the probability of an entity’s presence and its spatial attributes. The DigitCaps layer encodes high-level features, like the entity, where each capsule’s output is calculated using a weight matrix (M) and a component vector (X). A routing mechanism controls the connection between low-level and high-level capsules, updating the input vectors (V) for each capsule according to spatial relationships. Finally, the capsule outputs undergo the squash function, which ensures non-linearity and scales the values between 0 and 1, reflecting the activation level of each entity. The routing process iteratively fine-tunes the activation distribution until an optimal state is achieved, improving the network’s capacity to capture intricate spatial relationships in the input data.

### Extreme learning machine

The proposed study contributes to the highest classification accuracy by combining capsule networks for enhanced feature extraction. These features are then input into ELM, a type of neural network that has a single hidden layer and operates on auto tuning, as seen in Fig. 5. Extreme learning machines outperform other learning models due to their fast speed and low computational overhead. Among the learning models that are compared are random forest (RF), K-nearest neighborhood (KNN), support vector machines (SVMs), and the Bayesian classifier (BC). The neural network requires no tweaking and only employs one hidden layer. ELM uses a kernel function to generate accurate results for improved speed. Reduced training error and better approximation are two benefits of ELM that come from the auto tuning of weight biases and non-zero activation functions. The property of ELM is expressed mathematically by,

5 $$\begin{aligned} F_l(p) = \sum _{i=0}^{l} \alpha _i \, \text {hid}_i(p) = \text {hid}(p)\alpha \end{aligned}$$

Where,

p$$\rightarrow$$ input,

$\alpha \rightarrow$ output weight vector,

which are presented as follows:

6 $$\begin{aligned} \alpha = (\alpha 1, \alpha 2, \cdots ,\alpha \text {l})^{T} \end{aligned}$$

The hidden layer output, hid(p), is determined by Eq. (7).

7 $$\begin{aligned} hid(p)={h_1 (p),h_2 (p), \cdots h_l (p)} \end{aligned}$$

Therefore, Eq. (8) is used to get the result.

8 $$\begin{aligned} F_l(p) = \text {hid}(p)\alpha = \text {hid}(p) H^T \left( \frac{1}{C} HH^T \right) ^{-1}*O \end{aligned}$$

In this model, the output target vector is represented as O which are determined by the weights and bias of the hidden layer, which are calculated using Moore’s pseudo-inverse (MPI). Equation (8) is employed to classify the infectious consequences of COVID-19 on the lungs. The suggested network’s operating process is outlined in Method 1.

#### Parameters utilized in ELM

The parameter (p) input data is fed into a single hidden layer, with the output determined by the output weight vector ($\alpha$), which links the hidden layer to the final predictions. The activations of the hidden layer, denoted as hid(p), are computed using the ReLU activation function, with the output from each hidden unit contributing to the final result. ELM utilizes a Gaussian kernel function to enhance speed and accuracy by mapping the data into a higher-dimensional space, which facilitates more effective classification. It uses one-hot encoding to represent class labels and applies a softmax classifier for multiclass classification tasks. The model’s weight biases are automatically adjusted to reduce training error and enhance approximation. The output is calculated using Moore-Penrose pseudo-inverse, which approximates the matrix inverse to obtain a minimal-norm least-squares solution. This closed-form computation avoids iterative weight tuning, thereby reducing overfitting by preventing the model from memorizing noise in the data. It enhances generalizability by ensuring that the learned weights reflect the most stable and representative mapping from features to output. Furthermore, activation functions are applied in the hidden layer, allowing the network to model complex relationships within the data. Together, these parameters enable ELM to achieve lower computational costs, faster convergence, and robust classification performance.

#### Method 1: The algorithm for the recommended ensemble method


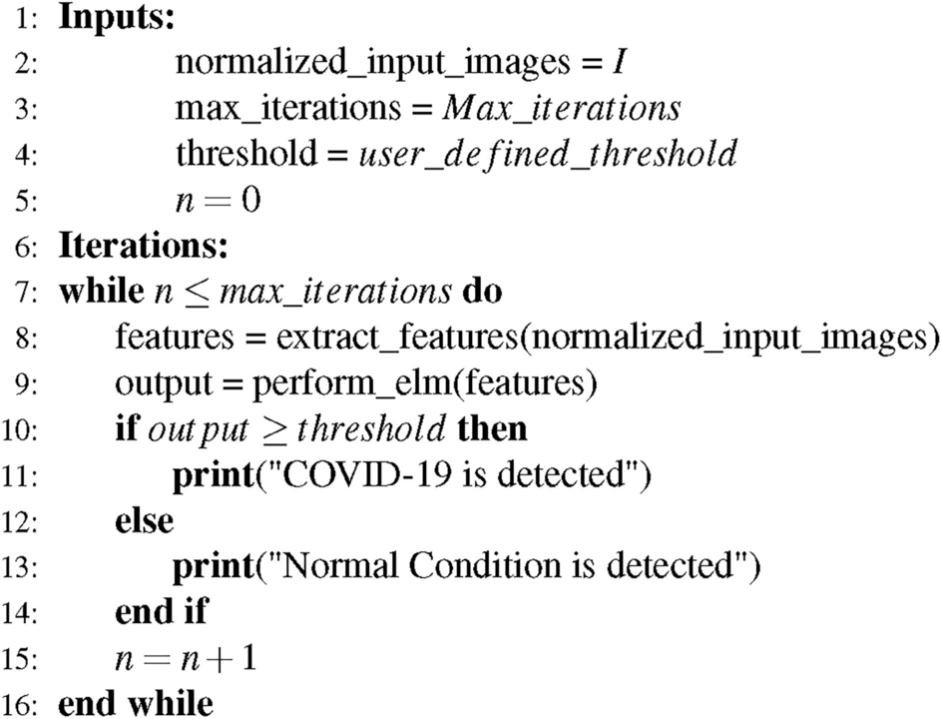


### Combined learning for global training

**Fig. 6:**
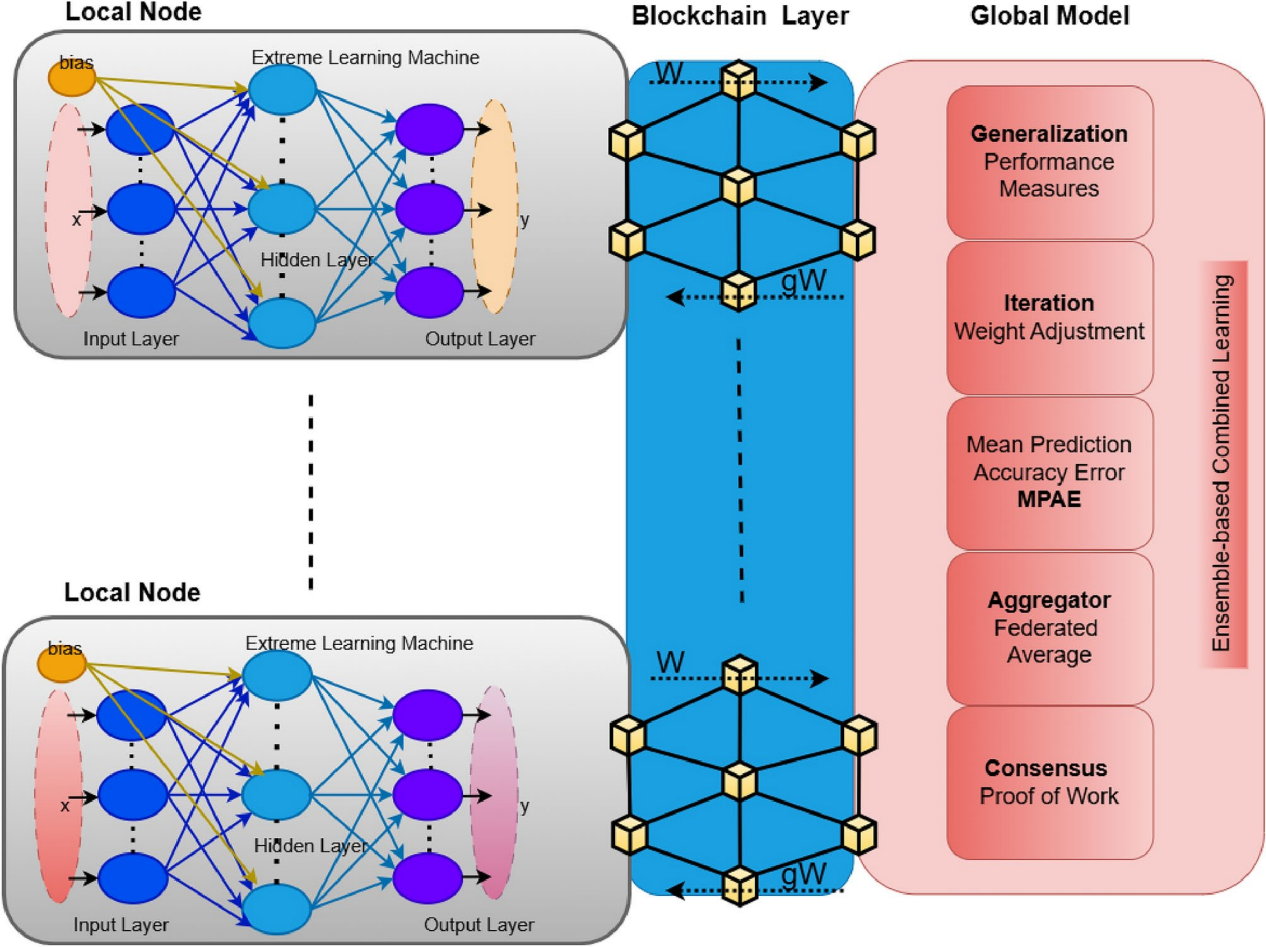
The suggested framework’s combined learning models were strengthened using blockchain technology.

In this segment, we look at a decentralized approach to data exchange across many hospitals. The proposed strategy leverages combined learning to disseminate hospital models collectively while maintaining privacy, combining many hospital models from various institutions. For the distributed training setup, five nodes were chosen based on preliminary experimentation, which indicated that this number provides a good balance between model performance, training efficiency, and communication cost. Fewer nodes reduced ensemble diversity, while more than five increased latency and overhead with negligible accuracy gain. In this case, DA stands for the total datasets and H for the number of hospitals. The global model M is regarded as the ensemble learning model in the suggested Combined model, and the ELM weights W are dispersed at random to different hospitals. Figure 6 depicts the combined learning model that was employed in this investigation. Hospitals use a overall or collective model, referred to as “Combined learning,” to combine weights from locally learned models. This is done by introducing a blockchain-based combined learning framework for collaborative training and sharing. In order to manage heterogeneous CT scan data, the first stage is gathering data from several sources into the local model and using a normalization technique.

After the data has been standardized, the following step is to train the model for segmenting visuals using the ensemble capsule network for COVID-19 diagnosis. Finally, to train a global model, the weights of the local model are distributed over the blockchain network. Let DA stand for all datasets (including training and testing datasets), and let HO stand for the number of hospitals, as determined by Eq. (9).

9 $$\begin{aligned} DA_i^{\text {train}} = \left\{ \left( X_{i,j}^{\text {train}}, Y_{i,j}^{\text {train}} \right) \right\} \end{aligned}$$

Where j =1 to N - training Data Additionally, testing results are included in Eq. (10).

10 $$\begin{aligned} DA_i^{\text {test}} = \left\{ \left( X_{i,j}^{\text {test}}, Y_{i,j}^{\text {test}} \right) \right\} \end{aligned}$$

Where j =1 to N - testing Data Equation (11) therefore represents the whole dataset that was utilized to train the global model.

11 $$\begin{aligned} DA(i) = DA_i^{\text {train}} \, UD_i^{\text {test}} \end{aligned}$$

The distribution of the data is still uneven as the hospitals are a varied source of the DA (i) data. During every communication cycle, the hospitals split up the ELM weights, or W. The hospitals use weights that are gathered and kept on the blockchain network to create a local model. After every round, weights are revised and uploaded to the blockchain network. The updated weights are numerically shown in Eq. (12).

12 $$\begin{aligned} \mu = UW^i - UW^1 \end{aligned}$$

Where $UW^i$ and $UW^1$ represents for the distributed weights of the local model and the global model. In the end, the proposed deep learning algorithm applies the newly created learning model, which is based on the ELM principle, derived from the combination of the block chain’s all-local models.

#### Parameters utilized in global model

The global model in combined learning involves several critical parameters and processes. The total datasets, represented as DA, encompass all the data gathered from the participating hospitals, while H denotes the number of hospitals involved in the decentralized framework. The global model (M) is an ensemble learning model created by aggregating local models from each hospital using Federated Averaging. The weights of the Extreme Learning Machine (ELM) classifier (W) are shared and updated across hospitals through a secure blockchain-based network. The data is divided into training ($DA^\text {train}$) and testing ($DA^\text {test}$) datasets, which are used to train and evaluate the global model, respectively, as outlined in Eqs. (9) and (10). The combination of these datasets into a global dataset DA(i) is represented by Eq. (11). During each communication cycle, with a block time, hospitals update and share their locally computed weights on the blockchain, including the block size. The differences in weights between the local and global models are represented by Eq. (12), where the weight updates ($\upmu$) are calculated. A normalization technique is employed to standardize the data from different hospitals, ensuring uniformity before training the models. The global model leverages an ensemble Capsule Network, which is used to segment CT scan images for COVID-19 diagnosis. This collaborative learning process allows the hospitals to improve their models collectively while preserving privacy, with each hospital benefiting from the aggregated knowledge without sharing their raw data.

#### Training the global model

The global model is developed using a collaborative learning approach, as shown in Fig. 6, which utilizes decentralized training across various hospitals. Each hospital independently trains its local model on its private dataset, utilizing Capsule Networks and Extreme Learning Machines (ELM) to suit their unique healthcare requirements. Hospitals ensure data privacy and security by refraining from sharing raw patient data. Instead, only the model weights are shared through a secure blockchain-based system. The blockchain enables secure aggregation of model updates, providing trust, transparency, and traceability throughout the sharing process. The global model is formed through an ensemble aggregation process, combining the model weights from all local models via a consensus-driven learning method supported by the blockchain. After the global model is developed, it is sent back to the hospitals. Each local model is then evaluated on new, unseen data, and its performance metrics are communicated to the global model. The global model calculates combined performance metrics, transforming into a more generalized and collaborative model that improves COVID-19 diagnosis across the network.

### Blockchain outline for combined learning

The suggested system integrates the blockchain architecture to provide secure and effective data transmission and retrieval for combined learning. Multiple hospitals working together can develop and refine the models to enhance disease identification. The research discusses the methods used in the data sharing and retrieval system that is based on the multiple-organization blockchain architecture.

#### Layered architecture of blockchain for clinical diagnosis

The proposed approach employs a stratified blockchain structure to ensure security, transparency, and cross-platform compatibility in clinical diagnostics. To facilitate modular operations and efficient connectivity with with Internet of Medical Things (IoMT) devices, the proposed architecture follows a multi-layered structure, comprising the following key components^55^.

- Application Layer: Facilitates interaction with healthcare applications such as diagnostic platforms, imaging workstations and medical record systems. It provides real-time feedback and interactive visualization of clinical insights.However, it is susceptible to security issues such as smart contract vulnerabilities, API misuse, and phishing-style UI attacks. To address these risks, the architecture incorporates rigorously audited smart contracts, multi-factor authentication (MFA), and robust access control mechanisms to ensure secure user interaction and prevent unauthorized access.
- Protocol Layer: Implements trusted algorithms to align decentralized nodes-such as Proof of Work (PoW)-ensuring consistency and immutability of healthcare data. Despite the potential for energy consumption and threats like 51% attacks, denial-of-service (DoS), or selfish mining, these risks are mitigated through carefully designed system parameters such as restricted node participation, adaptive difficulty levels, and real-time monitoring. In the context of clinical diagnosis, the trustworthiness and resilience offered by PoW outweigh its energy overhead, especially when deployed in a controlled, permissioned environment with limited but trusted nodes.
- Data Layer: Safeguards medical information such as scan results, health records, and predictive outputs using a tamper-resistant storage mechanism, while upholding transparency and traceability. Threats in this layer include unauthorized access, data manipulation, and ledger poisoning, which could compromise diagnostic accuracy. To mitigate these risks, the system enforces access control lists (ACLs), along with advanced cryptographic techniques like differential privacy and homomorphic encryption, enabling secure and privacy-preserving data sharing and analysis.
- Network Layer: Supports secure and efficient peer interactions, manages the discovery of participating nodes, and enables data sharing across medical infrastructure.It is exposed to various threats such as Sybil attacks, replay attacks, and man-in-the-middle (MITM) attacks that can compromise data in transit or disrupt communications. The proposed system employs TLS/SSL encryption, node authentication protocols, and timestamped transactions to preserve message integrity and prevent unauthorized data injection or eavesdropping.
- Physical Layer: Encompasses IoMT components such as sensors and diagnostic hardware, responsible for collecting patient data in real time and securely relaying it to subsequent layers. Security threats in this layer include sensor spoofing, hardware tampering, and side-channel attacks, which may lead to data manipulation at the source. To secure this layer, the system incorporates secure boot protocols, device-level authentication, and continuous anomaly detection to ensure only trusted devices participate and function reliably within the network.

**Fig. 7:**
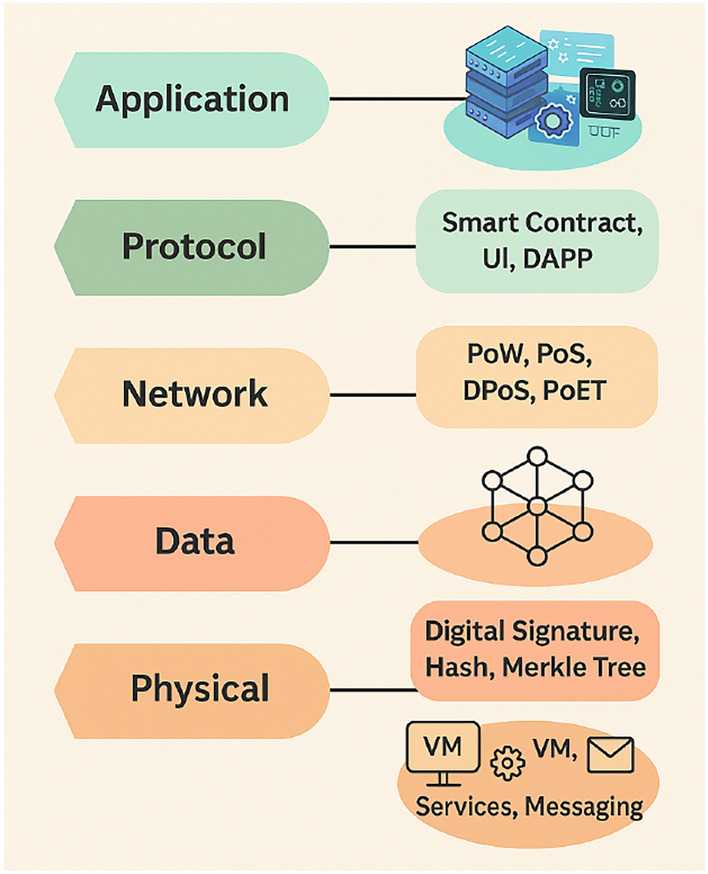
Layered architecture of blockchain.

The multi-layer design, as illustrated in Fig. 7, supports the secure implementation of blockchain-based diagnostic systems in healthcare environments, ensuring patient data privacy and promoting reliable inter-institutional collaboration^56^.

#### Data retrieval process using blockchain technology

Every hospital sources contributes the local model (data), which is then stored in the block chain network as a transaction. Two factors, the distance (d) between the hospital’s ID (ID) and the nodes, dictate how to retrieve the data from the nodes. An individual ID is generated based on the separation between the hospitals. The log tables containing the hospital’s unique ID are kept up to date with the help of the blockchain. The data are sourced from nearby hospitals, which you may recognize by their distinctive IDs. Hospitals are represented mathematically as X divided into several communities, where they are regarded as nodes. Equation (13) provides the formula needed to obtain the neighborhood distance between the nodes.

13 $$\begin{aligned} d(p(i), p(j)) = \sum _{a,b \in \mathbb {X}} \left\{ p(i), Up(j) - p(j), np(j) \right\} \end{aligned}$$

$$\begin{aligned} \text {Node Attributes} \rightarrow \sum _{a,b \in \mathbb {X}} \left( \{ p(i), Up(j) \} \right) \cdot \log (p(i), p(j)) \end{aligned}$$

Where the local model hospitals, designated by their distinct IDs, are p(i) and p(j), and are situated at the $i^\text {th}$ and $j^\text {th}$ positions, respectively. Next, the CLCD-Block model is trained utilizing the stored local models by means of the consensus technique. In this method, every node collaborates to train the CLCD-Block. It enables data transfer between several nodes by providing proof of work. Using the mean prediction accuracy error (MPAE), the consensus technique uses collaborative training to confirm that the local models are correct. The MPAE is the outcome of improved future prediction ability. To ensure data privacy, all of the node’s data are secured using wildly unpredictable, encryption techniques that employ public and private keys. MPAE reviews every transaction before it is put to the blockchain’s distributed ledger.

#### Blockchain-based data sharing process

The security element is crucial to the intricate process of data sharing across institutions, especially between requesters and source hospitals, according to the proposed structure. A safer alternative is suggested, in which hospitals only provide trained models to people who request them, as opposed to sharing all datasets as is now done. Hospitals must establish communication channels as part of the procedure, and a consensus mechanism is applied to facilitate learning from combined data. The development of models from many healthcare sources requires the application of collaborative learning. This approach is special because it uses blockchain technology, which provides a safe sanctuary for requesters’ and providers’ data inside blockchain nodes. Ensuring robust safeguards for data privacy is a crucial top priority. To do this, the strategy involves sharing just the learned models-not the original data. This ensures that sensitive data is protected and that the collaborative learning process is conducted without endangering the privacy of individuals. The recommended structure consists of two phases. During the first phase, all participating hospitals provide their imaging datasets. This collaborative effort builds the foundation for the second stage, which involves hospitals communicating the weights of the locally trained model with the blockchain framework. The global model is then created by combining the observations from each local model through the use of combined learning techniques.

## Experimental results

Unlike typical models, which were produced using TensorFlow version 1.8, the suggested model was created with TensorFlow Combined version 2.1.0. Keras is used by both models as their backend. The dataset is divided in a 70:20:10 ratio for training, testing, and validation to conduct the experiments effectively. TensorFlow Combined version 2.1.0 is used to train the model, which consist of four datasets containing 23,804, 31,501, 2,443 and 425,024 CT scan visuals, making up 70% of the dataset. Concurrently, TensorFlow version 1.8’s classical model is trained using these data. A CSS script powers the block chain’s user interface, while Python 3.9.1 is used to build the models on a unified blockchain. The approach is tested and validated with this configuration. Numerous performance indicators, including the F1-score, recall, specificity, accuracy, and precision, are used to evaluate the suggested design. The model’s capacity to distinguish between COVID-19 and non-COVID infections is evaluated by these measures. In a healthcare diagnosis system, it is essential to achieve high recall, precision, and accuracy. The output weights are calculated using the Moore-Penrose generalized inverse, which helps reduce overfitting and improves the model’s generalizability. When there is no improvement in validation performance following hyperparameter adjustments as shown in Table 3, this approach concludes the scheduled network training.

**Table 3:** Hyperparameter configuration of the proposed study.

Hyperparameters	Configuration
Local model	
Optimizer	Adam
Learning rate	0.01
Batches	32
Epochs	50
Hidden layers	01
Activation	ReLU
Kernal	Gaussian
Encoding	One-hot
Classifier	SoftMax
Error minimizer	Moore-Penrose pseudoinverse
Global model	
Consensus mechanism	Proof of Work (PoW)
Error minimizer	Mean Prediction Accuracy Error (MPAE)
Aggregation	Federated Averaging (FedAvg)
Block size	10MB
Block time	15 Sec

### Results and findings

**Fig. 8:**
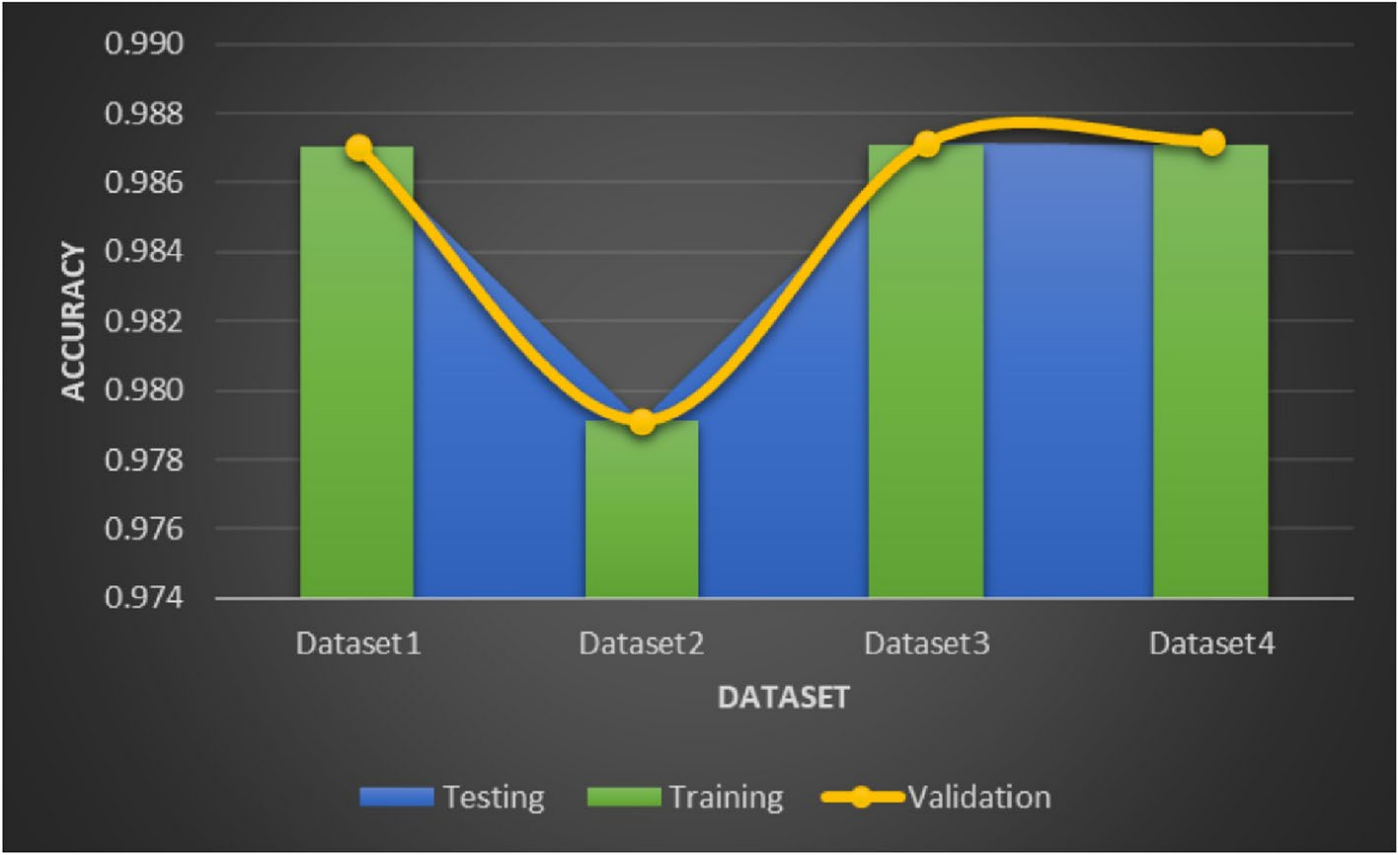
Error distribution plot demonstrating the accuracy of model performance.

**Fig. 9:**
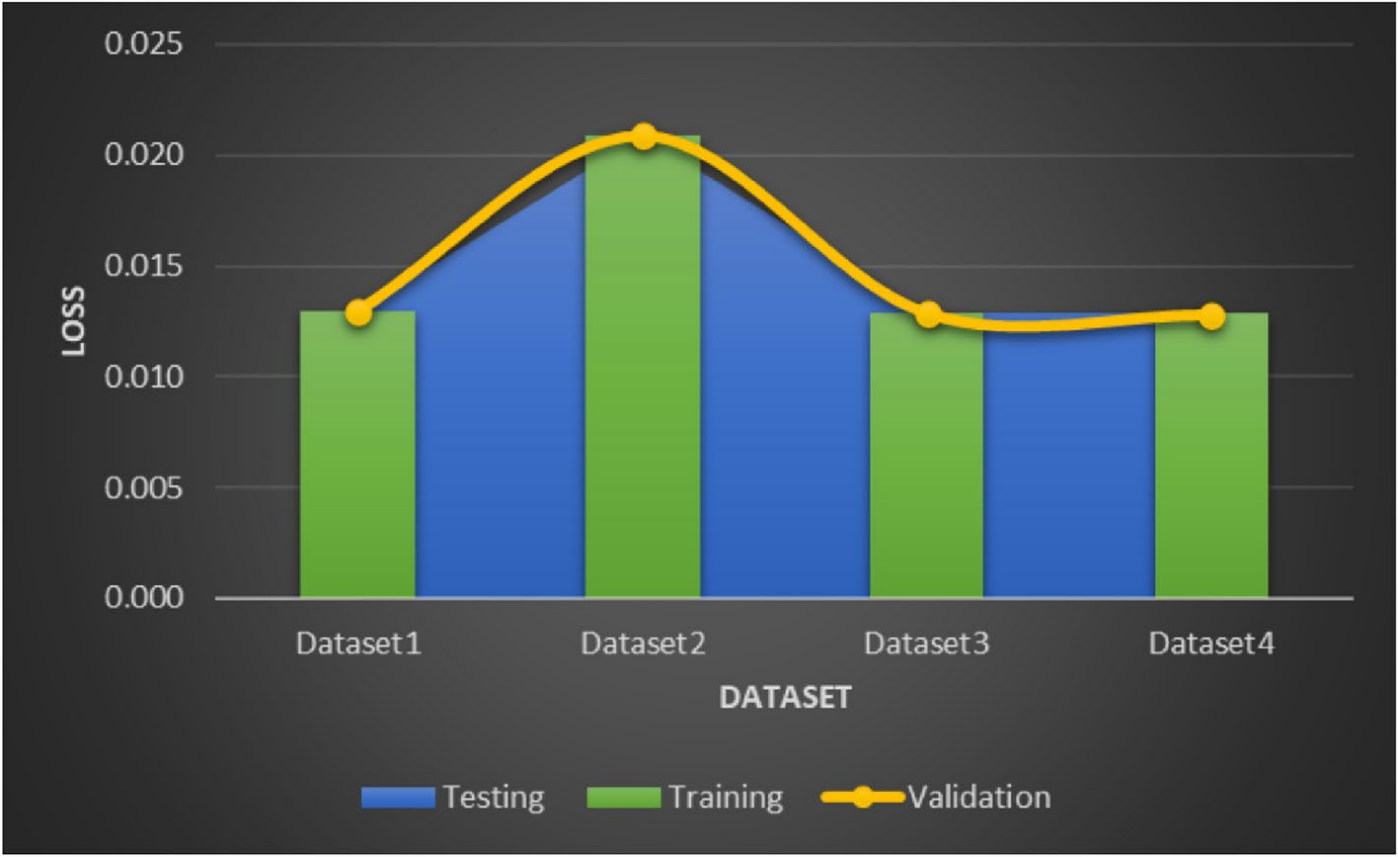
LVC of the proposed model for Datasets 1, 2, 3 and 4.

An assessment is conducted in three different aspects to evaluate the effectiveness of the proposed framework. Performance metrics for the suggested approach are calculated over a range of CT datasets in the first fold. Moreover, loss validation curves (LVC) are produced to support the suggested architecture’s performance. In the end, a comparison with various deep learning algorithms currently in use is used to show how excellent the suggested approach is. The error distribution plot in Fig. 8 demonstrates the accuracy of the suggested model’s performance across the datasets, while the LVC in Fig. 9 represents the loss function. A consistent pattern can be seen in both Figs. 8 and 9, The training and validation models are evaluated using the root mean square error (RMSE), which is measured at 0.0001. The validation curve for Dataset 2 indicates that the RMSE in Fig. 9 is marginally higher at 0.0021 due to the larger number of instances. Consequently, the proposed model’s overall performance in identifying COVID-19 for datasets 1, 2, 3 and 4 shows accuracy rates of 98.7%, 97.3%, 98.4% and 97.9%.

**Fig. 10:**
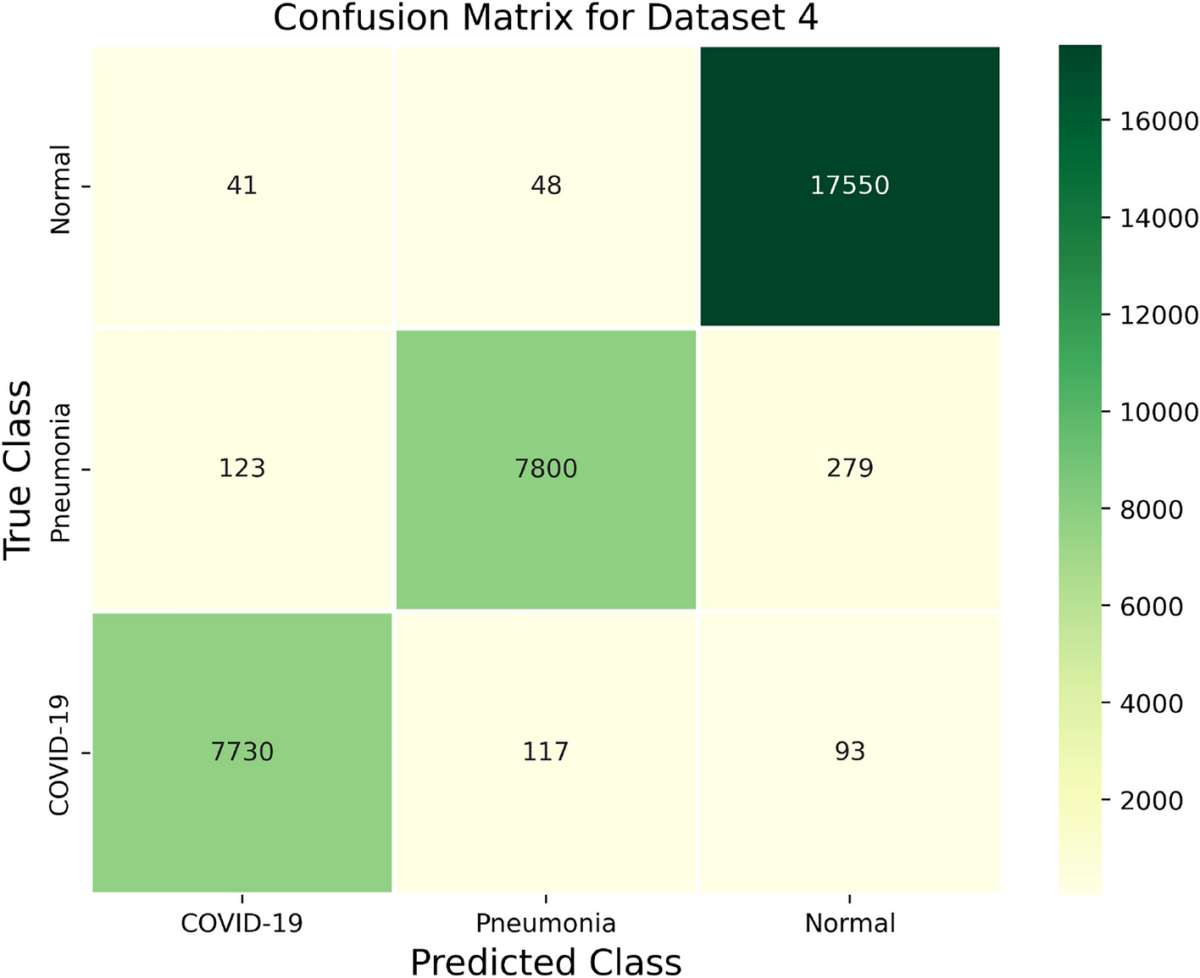
Multi-class confusion matrix of the proposed CLCD-block model for dataset 4.

The confusion matrix as shown in Fig. 10, summarizes the classification outcomes for three categories: Normal, Pneumonia, and COVID-19. The model demonstrated good performance, correctly identifying most instances within these categories. Out of the Normal instances, 17,550 were correctly identified, while 279 were misclassified as Pneumonia and 93 as COVID-19, suggesting some overlap in distinguishing Normal from these two conditions. The model accurately classified 7800 Pneumonia instances, but 48 were incorrectly labeled as Normal and 117 as COVID-19, indicating a small degree of overlap between Pneumonia and the other categories. Similarly, 7730 COVID-19 instances were correctly classified, though 41 were misclassified as Normal and 123 as Pneumonia, reflecting some challenge in distinguishing COVID-19 from the other categories. Overall, the model demonstrated strong performance, with minimal misclassifications, though some confusion between the Normal class and the two disease categories was observed.

**Fig. 11:**
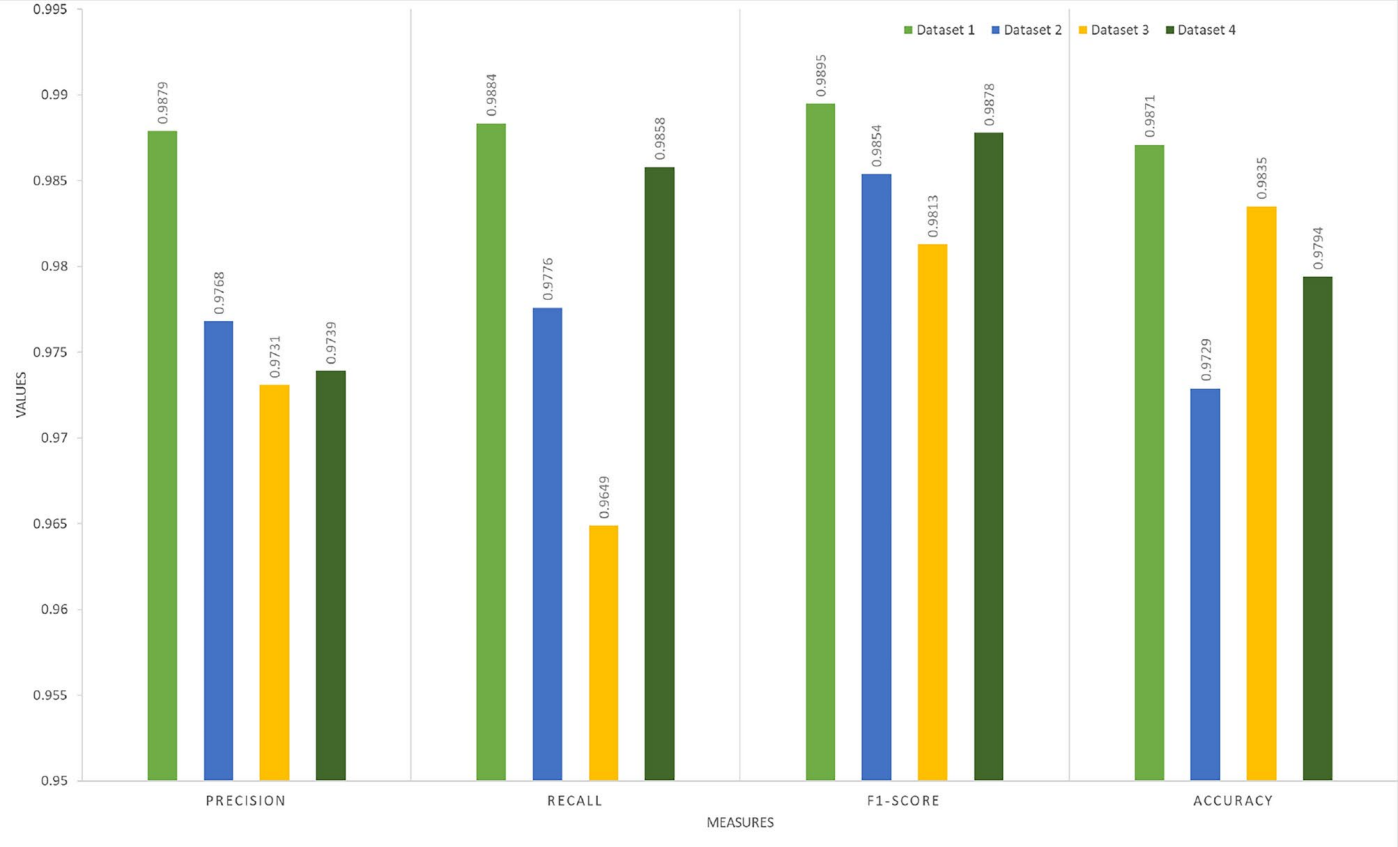
Performance evaluation of the proposed framework using various metrics across Datasets 1, 2, 3 and 4.

**Fig. 12:**
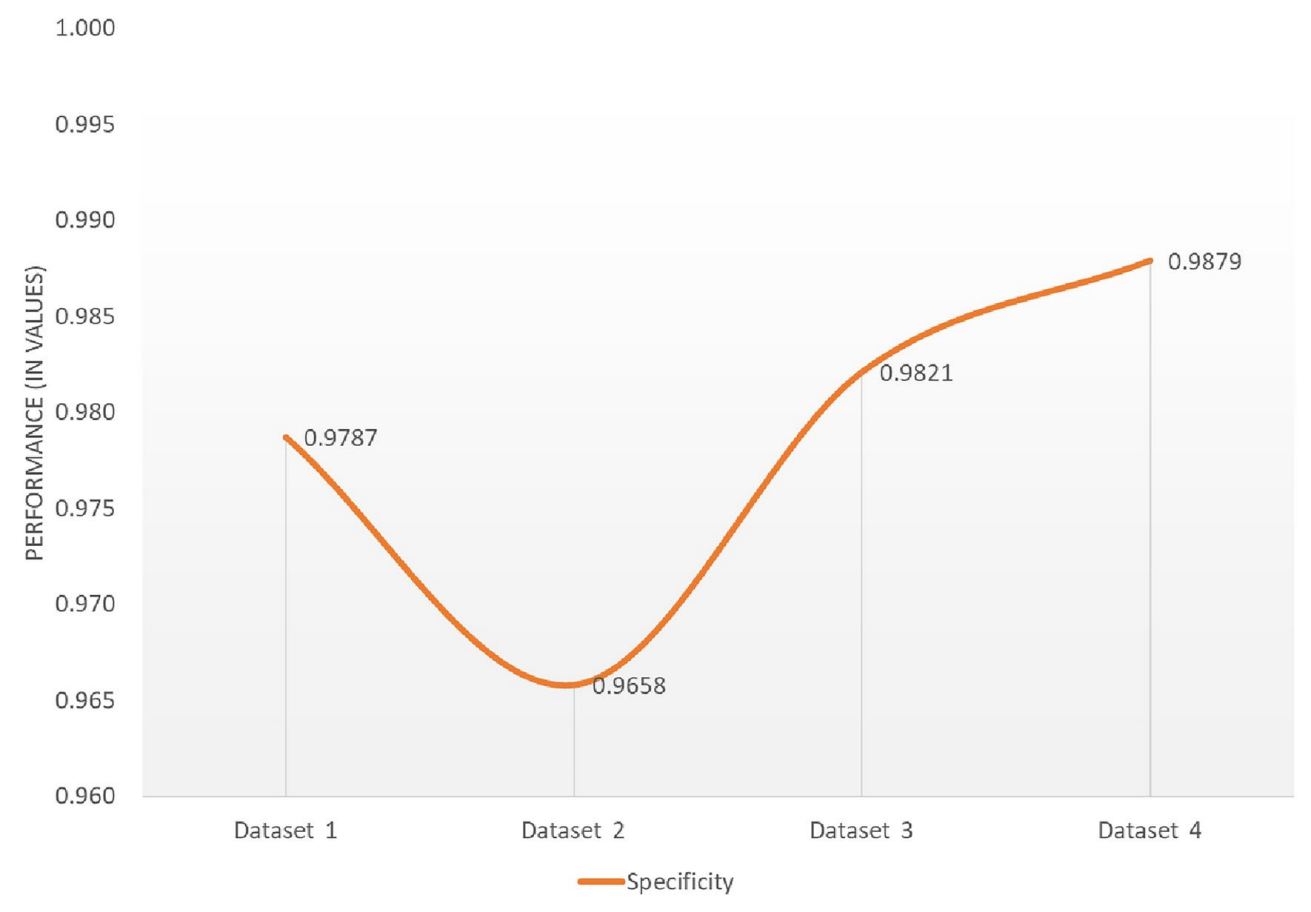
Performance evaluation of the proposed framework using Specificity metric across Datasets 1, 2, 3 and 4.

**Fig. 13:**
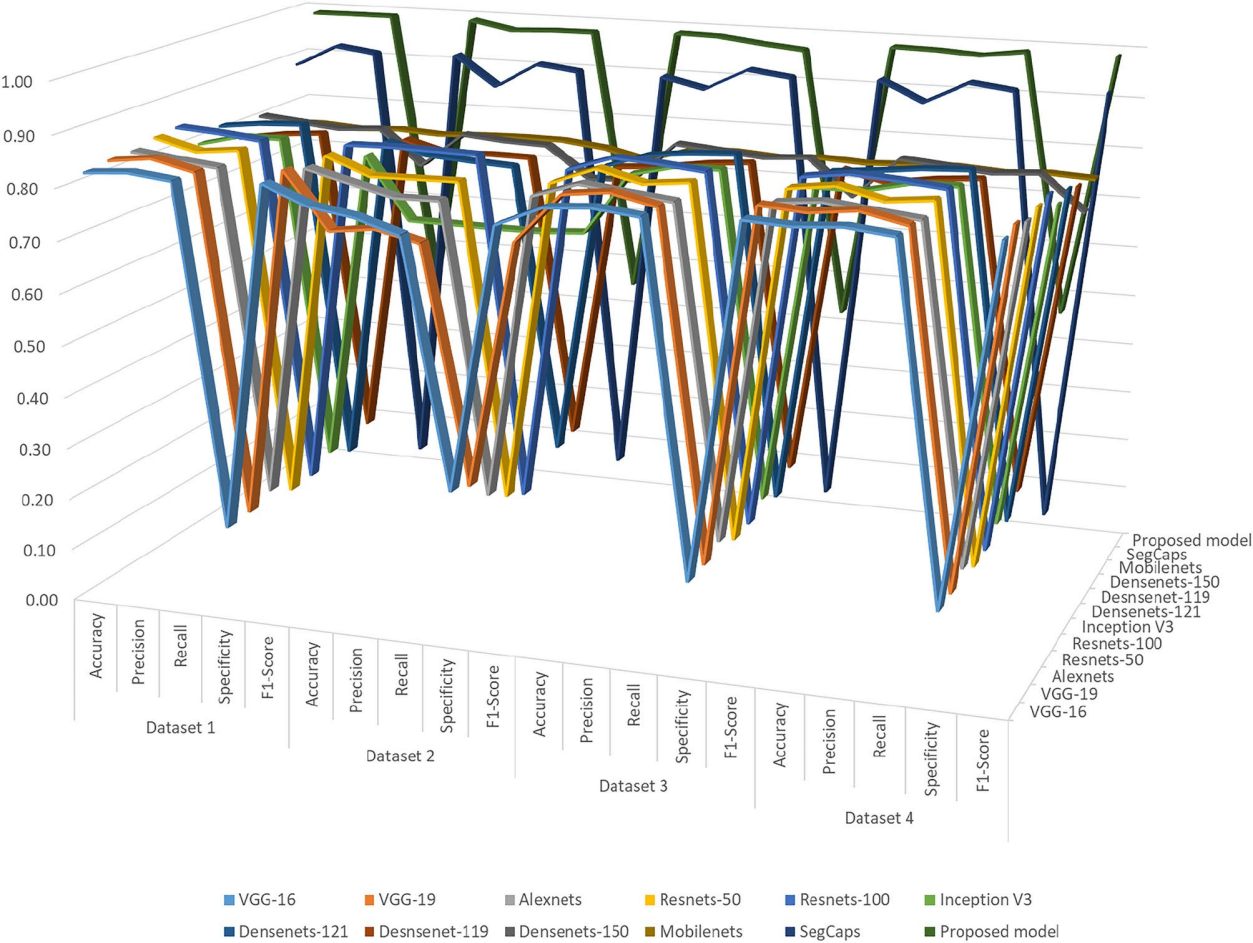
Comparison of the proposed CLCD-Block framework with baseline methods for COVID-19 detection across all the four Datasets.

**Table 4:** Comprehensive evaluation of the proposed model against existing frameworks for COVID-19 diagnosis across datasets 1, 2, 3, and 4.

Dataset	Models	Precision	Recall	Specificity	F1-Score	Accuracy
Dataset 1	EfficientNetB4^25^	0.9058	0.9258	0.9014	0.9157	0.9254
	Deep CNN^31^	0.8954	0.9152	0.8723	0.9052	0.9158
	Blockchain-basedfederated learning^38^	0.9258	0.9261	0.9154	0.9259	0.9174
	XGBoost^26^	0.9158	0.9014	0.8274	0.9085	0.9126
	CAP-CNN^41^	0.9314	0.9409	0.9026	0.9361	0.9417
	LSTM+blockchain^14^	0.9587	0.9414	0.8858	0.9500	0.9514
	Bi-LSTM+blockchain^33^	0.9458	0.9325	0.9087	0.9391	0.9418
	CLCD-Block (Proposed)	0.9879	0.9884	0.9787	0.9881	0.9871
Dataset 2	EfficientNetB4^25^	0.8852	0.8987	0.6547	0.8919	0.9021
	Deep CNN^31^	0.8987	0.9185	0.8452	0.9085	0.8971
	Blockchain-basedfederated learning^38^	0.9189	0.9085	0.4521	0.9137	0.9154
	XGBoost^26^	0.8947	0.8547	0.5214	0.8742	0.9142
	CAP-CNN^41^	0.8754	0.8689	0.7524	0.8721	0.8725
	LSTM+blockchain^14^	0.9125	0.9142	0.9014	0.9133	0.9147
	Bi-LSTM+blockchain^33^	0.9248	0.9187	0.8549	0.9217	0.9097
	CLCD-Block (Proposed)	0.9768	0.9776	0.9658	0.9772	0.9729
Dataset 3	EfficientNetB4^25^	0.9154	0.9024	0.8874	0.9089	0.9285
	Deep CNN^31^	0.8989	0.8958	0.8254	0.8973	0.9105
	Blockchain-basedfederated learning^38^	0.9198	0.9052	0.4291	0.9124	0.9125
	XGBoost^26^	0.8245	0.8147	0.8258	0.8196	0.8198
	CAP-CNN^41^	0.8958	0.8578	0.82531	0.8764	0.8874
	LSTM+blockchain^14^	0.9147	0.9058	0.8685	0.9102	0.9025
	Bi-LSTM+blockchain^33^	0.9258	0.9158	0.8978	0.9208	0.9154
	CLCD-Block (Proposed)	0.9731	0.9649	0.9821	0.9690	0.9835
Dataset 4	EfficientNetB4^25^	0.9154	0.8849	0.7584	0.8999	0.9058
	Deep CNN^31^	0.8799	0.8971	0.8542	0.8884	0.9254
	Blockchain-basedfederated learning^38^	0.9254	0.9230	0.8756	0.9242	0.9148
	XGBoost^26^	0.7587	0.7895	0.7258	0.7738	0.7689
	CAP-CNN^41^	0.8782	0.8687	0.8658	0.8734	0.8725
	LSTM+blockchain^14^	0.9158	0.9021	0.9098	0.9089	0.9029
	Bi-LSTM+blockchain^33^	0.9236	0.9317	0.9157	0.9276	0.9256
	CLCD-Block (Proposed)	0.9792	0.9731	0.9879	0.9761	0.9792

**Table 5:** Effectiveness comparison of the proposed framework with existing blockchain-enabled learning models for COVID-19 diagnosis across Datasets 1, 2, and 3.

	Number of cases	Average accuracy performance (%)	Trust level	Sharing and retrieval
Dataset - 1				
LSTM+blockchain^14^	Huge	95.14	Never	Never
Bi-LSTM+blockchain^33^	Huge	94.18	Never	Never
Blockchain-based federated learning^38^	Huge	91.74	Intermediate	Very Well
CLCD-Block (Proposed)	Huge	98.71	Huge	Very Well
Dataset - 2				
LSTM+blockchain^14^	Very Huge	91.47	Never	Never
Bi-LSTM+blockchain^33^	Very Huge	90.97	Never	Never
Blockchain-based federated learning^38^	Very Huge	91.54	Intermediate	Very Well
CLCD-Block (Proposed)	Very Huge	97.29	Huge	Very Well
Dataset - 3				
LSTM+blockchain^14^	Huge	90.25	Never	Never
Bi-LSTM+blockchain^33^	Huge	91.54	Never	Never
Blockchain-based federated learning^38^	Huge	91.25	Intermediate	Very Well
CLCD-Block (Proposed)	Huge	98.35	Huge	Very Well
Dataset - 4				
LSTM+blockchain^14^	Very Huge	90.29	Never	Never
Bi-LSTM+blockchain^33^	Very Huge	92.56	Never	Never
Blockchain-based federated learning^38^	Very Huge	91.48	Intermediate	Very Well
CLCD-Block (Proposed)	Very Huge	97.92	Huge	Very Well

The unique properties of ELM combined with capsule feature extraction, which is used as the classification layer in the proposed model, enable consistent performance over a wide range of datasets from many sources. Figure 11 displays the evaluation results of the proposed model across the four datasets. The findings indicate that the model’s performance falls between 0.9729 and 0.9871 for accuracy, 0.9731 and 0.9879 for precision, and 0.9649 and 0.9884 for recall. In all four datasets, as illustrated in Fig. 12, the proposed CLCD-Block framework achieved superior specificity across all evaluated datasets, with values of 0.9787 (Dataset 1), 0.9658 (Dataset 2), 0.9821 (Dataset 3) and 0.9879 (Dataset 4). These improvements reflect a significant reduction in false positives compared to baseline models, underscoring the model’s reliability in real-world deployment.

Figure 13 presents the various models’ performance alongside the recommended model, focusing on all four datasets. As the number of records increases in Dataset 2, all existing deep learning models show a decline in detection effectiveness. However, there is a remarkable trend that evidences the proposed framework’s superior performance compared to the other benchmarked models, with a significant increase when compared to SegCAP, ResNet-100. Consistently observed, this trend is similar to the one displayed in Fig. 13, which attests to the recommended model’s constant superior performance over other models, provides additional evidence for this outcome. Across several datasets, the proposed model, which combines ELM with capsule networks, achieves the highest accuracy in COVID-19 identification. This outcome proves the model’s superiority over alternative algorithms. In addition, a comparative analysis is conducted between the suggested model and current blockchain-based learning models, focusing on the amount of datasets, degree of trust, Precision, Recall, Specificity, F1-Score and Accuracy of detection.

The performance comparison Table 4, demonstrates the comprehensive evaluation of the proposed model against existing Blockchain-enabled learning frameworks for COVID-19 diagnosis across Datasets 1, 2, 3, and 4. It evaluates multiple models, including the LSTM+blockchain^14^, Bi-LSTM+blockchain^33^, Blockchain-based federated learning^38^ and our proposed CLCD-Block. These models are assessed across four datasets using key metrics such as precision, recall, specificity, F1-score, and accuracy. The proposed Combined Collective Capsule Networks exhibited consistently superior performance across all datasets, achieving higher precision, recall, F1-score, and accuracy compared to other models. This demonstrates the model’s ability to handle the specific challenges associated with the tasks, ensuring high performance and reliability. While the proposed model demonstrated better specificity than the others, all models showed suboptimal specificity, highlighting the need for further refinement to minimize false positives. The LSTM+blockchain performed reasonably well, exhibiting marginally improved precision and recall on Dataset 1. However, its specificity was notably lower when compared to the proposed model. Like the LSTM+blockchain, the Blockchain-based federated learning showed strong accuracy but fell short of the proposed model in terms of precision, recall, and F1-score. Additionally, its specificity was the lowest, suggesting a reduced capacity to accurately identify true negatives. The Blockchain-based federated learning yielded promising results, especially on Dataset 1 and Dataset 2, but its overall accuracy and performance were still outpaced by the proposed model. The results of this comparative assessment are compiled in Tables 4 and 5, which also provides details on the data sharing and retrieval capabilities of blockchain-based learning models. Collectively, Tables 4 and 5 shows the design and effectiveness of how well the proposed framework integrates with the blockchain architecture. In terms of trust level, detection accuracy, and dataset management, it shows promise. This placement demonstrates how effectively the recommended approach performs in enhancing blockchain data interchange and retrieval processes while upholding the most crucial data security and privacy regulations.

### Ablation study on CLCD-block model

To validate the effectiveness of each module within the proposed CLCD-Block architecture, we conducted an ablation study by selectively removing key components such as the collective learning mechanism, blockchain-enabled validation layer, and deep ensemble integration. As shown in Table 6, the complete CLCD-Block model achieves the highest performance across all metrics. Removing the blockchain layer led to a noticeable drop in accuracy (from 98.62% to 96.47%), highlighting its role in ensuring data integrity and trust. Excluding the collective learning component further reduced accuracy to 94.36%, indicating the importance of knowledge aggregation from multiple nodes. Similarly, removing the deep ensemble resulted in a performance decline to 92.14%. The lowest performance (89.87%) was observed when all enhancements were removed, confirming that each module contributes significantly to the model’s overall diagnostic capability.

**Table 6:** Ablation study on CLCD-Block model.

Model variant	Collective learning	Blockchain layer	Deep ensemble	Accuracy (%)	Precision (%)	Recall (%)	F1-Score (%)
CLCD-Block	Yes	Yes	Yes	98.71	98.79	98.84	98.81
w/o Blockchain Layer	Yes	No	Yes	96.47	96.12	96.85	96.48
w/o Collective Learning	No	No	Yes	94.36	94.20	94.60	94.39
w/o Deep Ensemble	Yes	Yes	No	92.14	91.90	92.50	92.20
Only Individual Base Model	No	No	No	89.87	89.45	90.10	89.77

### Statistical validation of proposed CLCD-Block model

To rigorously assess the performance improvement of the proposed CLCD-Block model over existing baseline models, a Wilcoxon signed-rank test was conducted. This non-parametric statistical test evaluates whether there is a significant difference in the paired metric scores (accuracy, precision, recall, F1-score, and specificity) across different models evaluated on the same datasets. Table 7 presents the p-values obtained from the Wilcoxon signed-rank test between CLCD-Block and each baseline model. The test was performed under the null hypothesis (H0) that there is no significant difference in the performance metrics between the models. The results demonstrate that all p-values are well below the 0.05 significance threshold, indicating that the null hypothesis can be confidently rejected. In particular, extremely low p-values were observed in comparisons with models such as EfficientNetB4 ($p = 2.38 \times 10^{-9}$) and Deep CNN ($p = 1.65 \times 10^{-8}$), underscoring the substantial and consistent performance gains achieved by the proposed method. Even in comparisons where performance gaps were narrower, such as Bi-LSTM + Blockchain ($p = 1.42 \times 10^{-4}$), the improvements were statistically significant. These findings confirm that the improvements achieved by CLCD-Block are not due to random variation but are statistically robust across all baseline comparisons. Hence, the proposed approach offers a significant advancement in diagnostic accuracy, reliability, and generalization.

**Table 7:** Statistical comparison of CLCD-Block vs. existing models.

Comparison model	* p*-value (Wilcoxon Test)	Significance (*p* < 0.05)	Implication
EfficientNetB4^25^	$2.38 \times 10^{-9}$	Significant	CLCD-Block significantly outperforms in all metrics
Deep CNN^31^	$1.647 \times 10^{-8}$	Significant	Consistently better results across datasets
Blockchain-based FL^38^	$7.372 \times 10^{-8}$	Significant	Superior robustness and diagnostic precision
XGBoost^26^	$1.4497 \times 10^{-7}$	Significant	Outperforms especially in recall and specificity
CAP-CNN^41^	$1.54972 \times 10^{-6}$	Significant	More generalizable and accurate detection
LSTM + Blockchain^14^	$2.000163 \times 10^{-5}$	Significant	Better recall and overall balance in performance
Bi-LSTM + Blockchain^33^	$1.42 \times 10^{-4}$	Significant	Marginal but significant improvement in F1 and accuracy

### Complexity analysis of the proposed study

The temporal and spatial complexities of the proposed framework are extensively contrasted with those of existing state-of-the-art models. The temporal complexity is evaluated using Big-O notation, often expressed as O(n). Given that traditional learning models operate on centralized systems, it is typical for the number of computations (N) to expand exponentially. On the other hand, the proposed model adopts a distributed method to minimize execution durations, depending on the number of nodes employed for training. By employing five nodes for training in our tests, we were able to reduce N to five. To assess hardware dependency, we further evaluated the proposed model on three different computational setups: (i) high-end GPU-enabled workstation (NVIDIA RTX 3090, 64 GB RAM), (ii) mid-range system (Intel i7 CPU, 16 GB RAM), and (iii) resource-constrained environment (Intel i5 CPU, 8 GB RAM). Across all configurations, the model consistently demonstrated efficient performance, with execution times for binary classification ranging from 0.218 seconds on high-end systems to 0.295 seconds on low-resource machines. This minimal variation confirms that the model’s efficiency is hardware-agnostic and suitable for deployment in diverse computing environments.Table 8 displays a comparison of the temporal and spatial complexity of the proposed framework with other benchmarked approaches, clearly showing that the proposed model exceeds the performance of all other models in terms of both time and space complexity. The temporal complexity variation of several algorithms is displayed in Table 8, demonstrating that the blockchain-enabled combined learning model is simpler than the benchmarked models because of its decentralized architecture. Space complexity, which quantifies the amount of memory needed for the model to function optimally, is another crucial consideration. The approach employs a measure of space complexity as memory. The Combined approach’s distributed design, which utilizes less memory, is helpful in this situation. Moreover, by including an extreme learning machine, the proposed combined model reduces space complexity even further because it is feed-forward. Our model demonstrates the best time complexity performance, with execution times of 0.218 seconds for binary classification and 0.312 seconds for multi-class classification, showcasing its superior efficiency over other models. The Blockchain-based federated learning takes 0.546 seconds for binary classification, while traditional models such as the Bi-LSTM+blockchain and LSTM+blockchain have notably higher times, reaching 0.725 seconds and 0.895 seconds, respectively. In terms of space complexity, our model excels with memory usage of 2.46 MB for binary classification and 3.21 MB for multi-class, significantly reducing memory consumption compared to the Blockchain-based federated learning 3.25 MB and traditional models, which require 5.54 MB and 6.92 MB, respectively. These findings emphasize the effectiveness of our proposed model, positioning it as an ideal solution for real-time applications in environments with limited resources, like healthcare diagnostics.

**Table 8:** Comparison of temporal and spatial complexity of the proposed model with that of existing blockchain models.

Models in blockchain	Time complexity (Sec)		Space complexity (MB)	
	Binary class	Multi-class	Binary class	Multi-class
LSTM+blockchain^14^	0.895	1.254	6.92	8.89
Bi-LSTM+blockchain^33^	0.725	0.962	5.54	6.98
Blockchain-based federated learning^38^	0.546	0.823	3.25	4.29
CLCD-Block (Proposed)	0.218	0.312	2.46	3.21

### Discussion and limitations of the proposed study

The proposed CLCD-Block framework demonstrates superior performance across four CT scan-based COVID-19 datasets, outperforming classical deep learning and blockchain-enabled models in accuracy, recall, precision, and F1-score. Integrating Capsule Networks with Extreme Learning Machines (ELM) enabled effective feature extraction and classification, with performance metrics consistently above 97% and RMSE values as low as 0.0001. Despite minor misclassifications observed in the confusion matrix-particularly among Normal, Pneumonia, and COVID-19 categories-the model maintains high detection reliability. The CLCD-Block inherently addresses dataset imbalance through Capsule Networks’ dynamic routing and ELM’s generalizable learning, ensuring fair class-wise performance without explicit balancing strategies. A comparative analysis further validates its robustness, with the CLCD-Block achieving higher metrics than LSTM+Blockchain, Blockchain-based federated learning, and others, particularly in recall and accuracy. The blockchain-enabled structure also strengthens data integrity, privacy, and trust, while the distributed training approach effectively reduces temporal and spatial complexity. With a time complexity of 0.218 seconds for binary classification and minimal memory usage (2.46 MB), the framework proves efficient and scalable. A brief scalability assessment was conducted by varying the number of nodes (3, 5 and 7). The model exhibited stable accuracy across configurations, with five nodes offering the optimal balance between learning diversity and communication efficiency. Adding more nodes led to marginal performance gains but increased system complexity and synchronization delays. An ablation study revealed that each component-the blockchain layer, collective learning, and deep ensemble-significantly contributes to overall performance, with notable drops in accuracy upon removal. Statistical validation using the Wilcoxon signed-rank test confirmed the significance of these gains (all p-values < 0.05), reinforcing that improvements are not random but statistically robust. False positive reduction is a critical factor in cybersecurity diagnostics. The CLCD-Block’s model design significantly enhances specificity by minimizing misclassifications. Additionally, the integrated blockchain structure supports distributed verification, further reducing false alerts. While specificity remains an area for refinement, the proposed model offers a powerful solution for rapid, secure, and reliable COVID-19 diagnosis. Its adaptability across diverse datasets and blockchain integration confirms its potential for real-world healthcare applications.

Despite its advantages, integrating blockchain into healthcare presents several challenges, such as scalability, latency, and data interoperability. High computational costs and the immutable nature of blockchain may also conflict with privacy regulations like GDPR. Moreover, real-time processing constraints can hinder clinical applicability. In time-critical clinical environments, latency may lead to delays in diagnostic decision-making or treatment initiation, underscoring the need for streamlined consensus and off-chain solutions. These issues can be mitigated through permissioned blockchains, lightweight consensus mechanisms, and hybrid models that use off-chain data storage for sensitive records. Adoption of interoperability standards like HL7 FHIR can enhance seamless data exchange. By addressing these concerns, blockchain can be more effectively aligned with the dynamic requirements of healthcare diagnostics and data sharing.

This study showcases significant progress, though there remain aspects that could benefit from further improvement. The model demonstrates effective scalability across different datasets, though its performance may vary with very large data sizes, suggesting areas for future refinement. Integrating blockchain improves security and trust, though tackling latency challenges could further strengthen its application in real-time clinical settings. Despite integrating blockchain, the model maintains real-time efficiency, as evidenced by low execution times and minimal memory usage. This confirms that the added security does not compromise computational performance. While the model demonstrates strong memory efficiency, further refinement could optimize resource utilization even more. Finally, adapting the model to accommodate larger healthcare multi-class datasets and improving cost-efficiency will broaden its applicability, further leveraging the solid foundation laid by this study. Despite integrating blockchain, the model maintains real-time efficiency, as evidenced by low execution times and minimal memory usage. This confirms that the added security does not compromise computational performance.

## Conclusion and future directions

The proposed CLCD-Block framework offers a comprehensive and effective solution to existing challenges in AI-based medical diagnosis, particularly for COVID-19 detection from CT scans. It integrates Capsule Networks with Extreme Learning Machines (ELM) for robust feature extraction and classification, consistently achieving performance metrics above 97% with RMSE as low as 0.0001 across four diverse datasets. Our model demonstrates high specificity, confirming its strength in minimizing false positives and enhancing deployment readiness. The inclusion of blockchain ensures data integrity, privacy, and trust during distributed training across hospitals. Ablation studies confirm the significant contribution of each module-collective learning, blockchain validation, and deep ensemble-in achieving superior accuracy. Statistical validation using the Wilcoxon signed-rank test demonstrates that the model’s improvements are significant and consistent compared to baseline models. With high efficiency (0.218 sec inference time, 2.46 MB memory usage) and adaptability, CLCD-Block proves to be a scalable and secure diagnostic tool suitable for real-world healthcare applications. The proposed CLCD-Block framework, though specifically designed for COVID-19, is versatile enough for diagnosing chronic diseases, managing global health emergencies, and facilitating secure data sharing while preserving privacy. Its adaptability ensures relevance to emerging and re-emerging diseases as well as other critical healthcare scenarios.

While the enhanced performance of the proposed strategy is acknowledged, further refinement is needed to adapt it for diverse real-time clinical datasets. Future work will focus on enhancing scalability by minimizing blockchain latency, optimizing computational efficiency, and improving cost-effectiveness. Additionally, efforts will be directed toward empirical evaluation using larger, heterogeneous, and multi-class healthcare datasets to demonstrate the model’s generalizability and operational readiness across broader diagnostic domains. Real-world pilot implementations in collaboration with healthcare institutions will also be pursued to assess clinical feasibility, regulatory compliance, and workflow integration. These directions aim to solidify CLCD-Block’s potential as a trustworthy and future-ready diagnostic platform in intelligent healthcare systems.

## Data Availability

The datasets used and/or analysed during the current study are available from the corresponding author upon reasonable request.
